# Supplementary material for: Genomic landscape associated with potential response to anti-CTLA-4 treatment in cancers
Source: Nat Commun. 2017 Oct 19;8:1050. doi: 10.1038/s41467-017-01018-0 (PMC5648801; doi:10.1038/s41467-017-01018-0)
Supplement: Supplementary file 1 — Supplementary Information [file 41467_2017_1018_MOESM1_ESM.pdf]

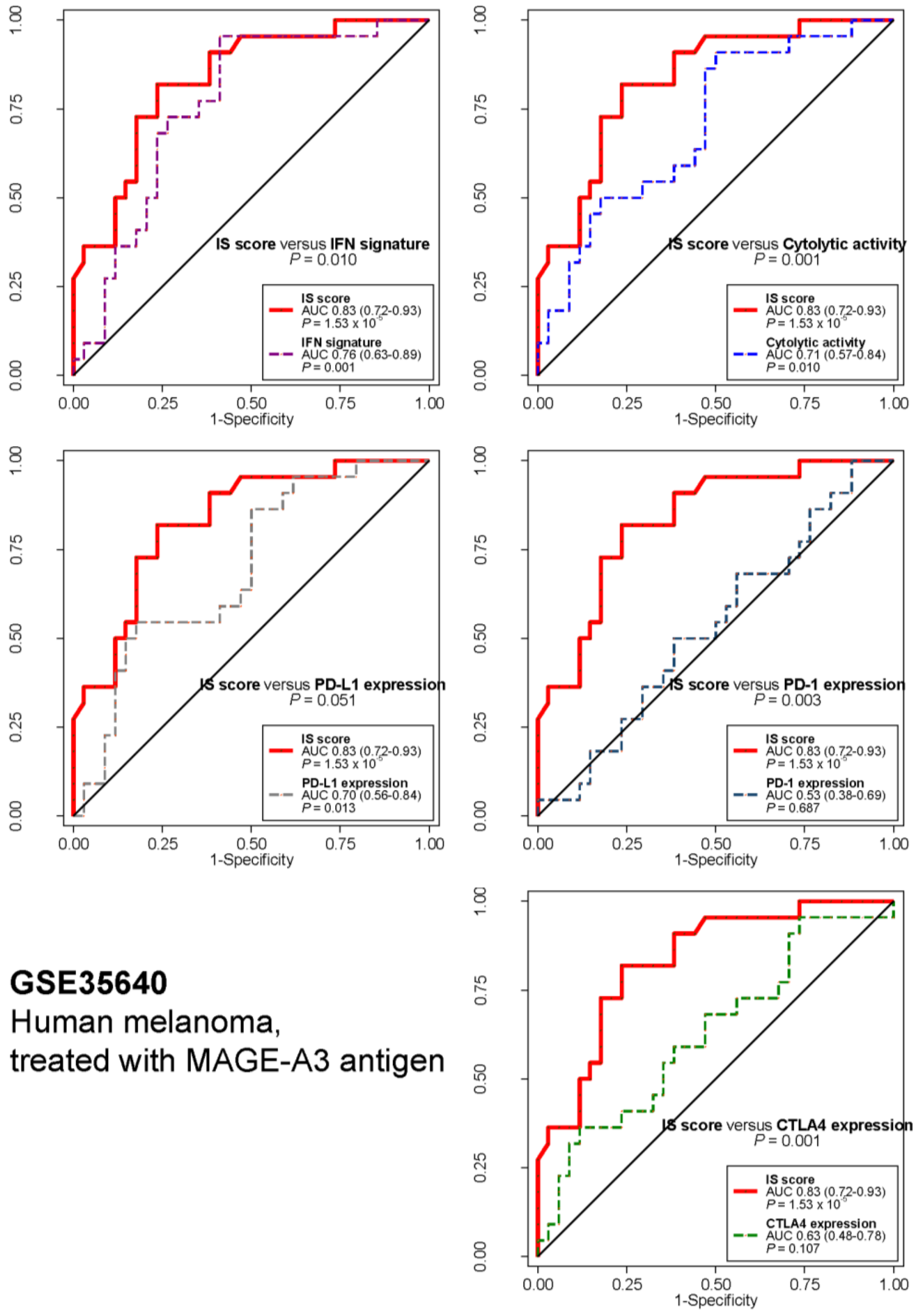

Supplementary Figure 1. Prognostic significance of immune signature (IS) score in human melanoma treated with MAGE-A3 antigen

Receiver operating characteristics (ROC) analysis of various immune biomarkers as well as IS scores from each prediction (top left, interferon-gamma (IFN) signature; top right, cytolytic activity; middle left, PD-L1 expression; middle right, PD-1 expression; bottom, CTLA-4 expression). Significance of IS biomarkers was estimated by area under curve (AUC) from ROC analysis.

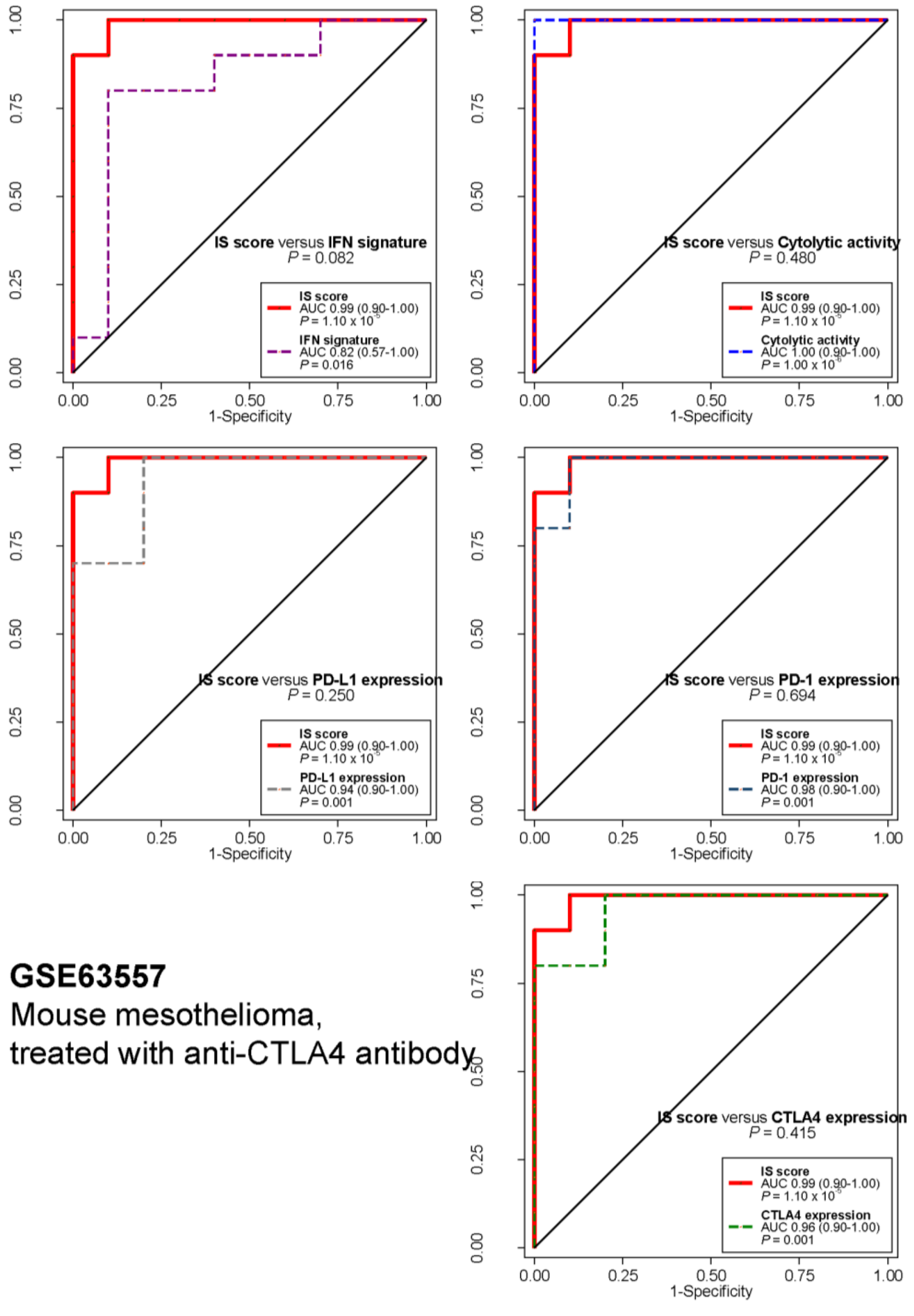

Supplementary Figure 2. Prognostic significance of immune signature (IS) score in mouse mesothelioma model treated with anti-CTLA-4 antibody

Receiver operating characteristics (ROC) analysis of various immune biomarkers as well as IS scores from each prediction (top left, interferon-gamma (IFN) signature; top right, cytolytic activity; middle left, PD-L1 expression; middle right, PD-1 expression; bottom, CTLA-4 expression). Significance of IS biomarkers was estimated by area under curve (AUC) from ROC analysis.

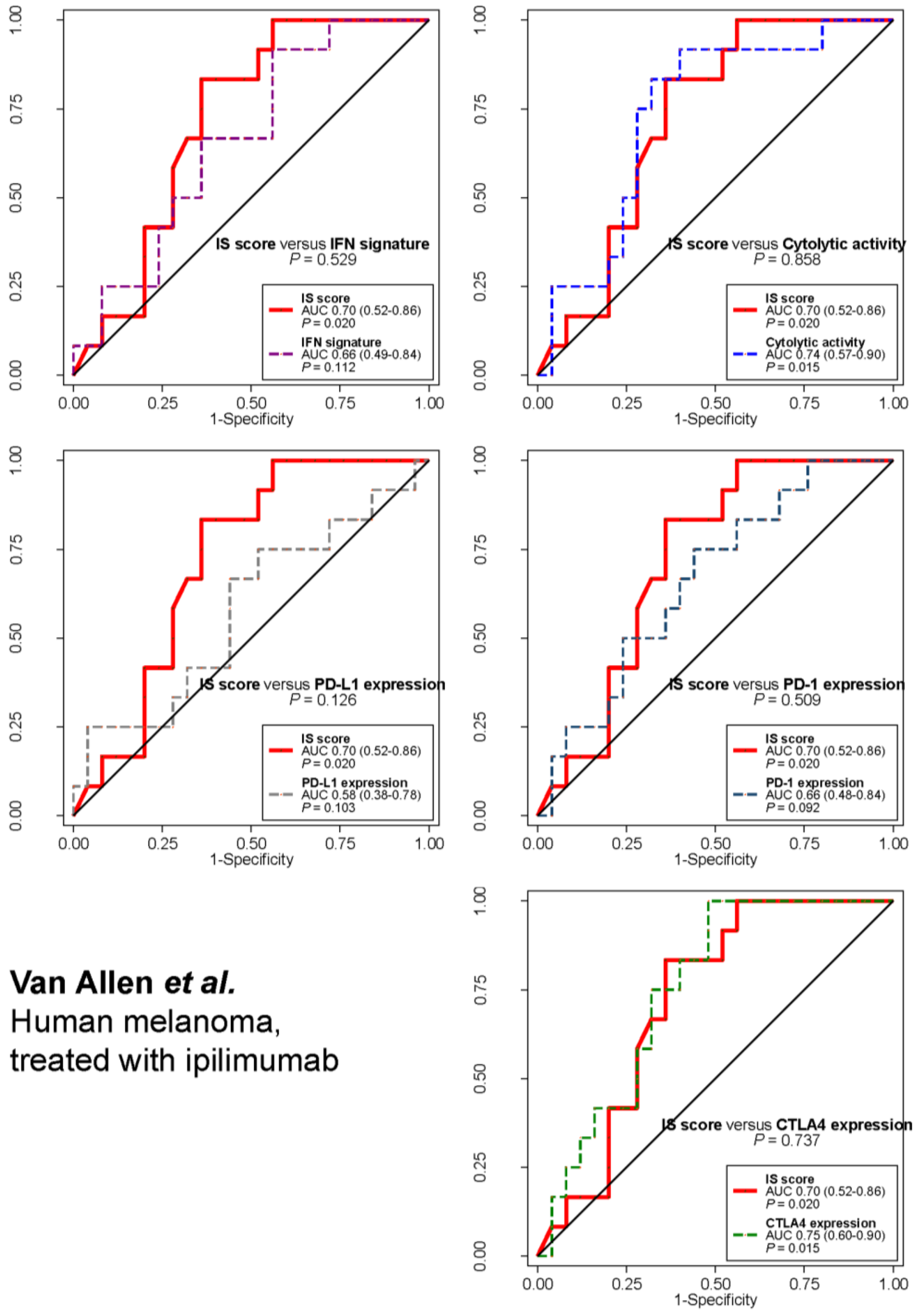

Supplementary Figure 3. Prognostic significance of immune signature (IS) score in human melanoma treated with ipilimumab

Receiver operating characteristics (ROC) analysis of various immune biomarkers as well as IS scores from each prediction (top left, interferon-gamma (IFN) signature; top right, cytolytic activity; middle left, PD-L1 expression; middle right, PD-1 expression; bottom, CTLA-4 expression). Significance of IS biomarkers was estimated by area under curve (AUC) from ROC analysis.

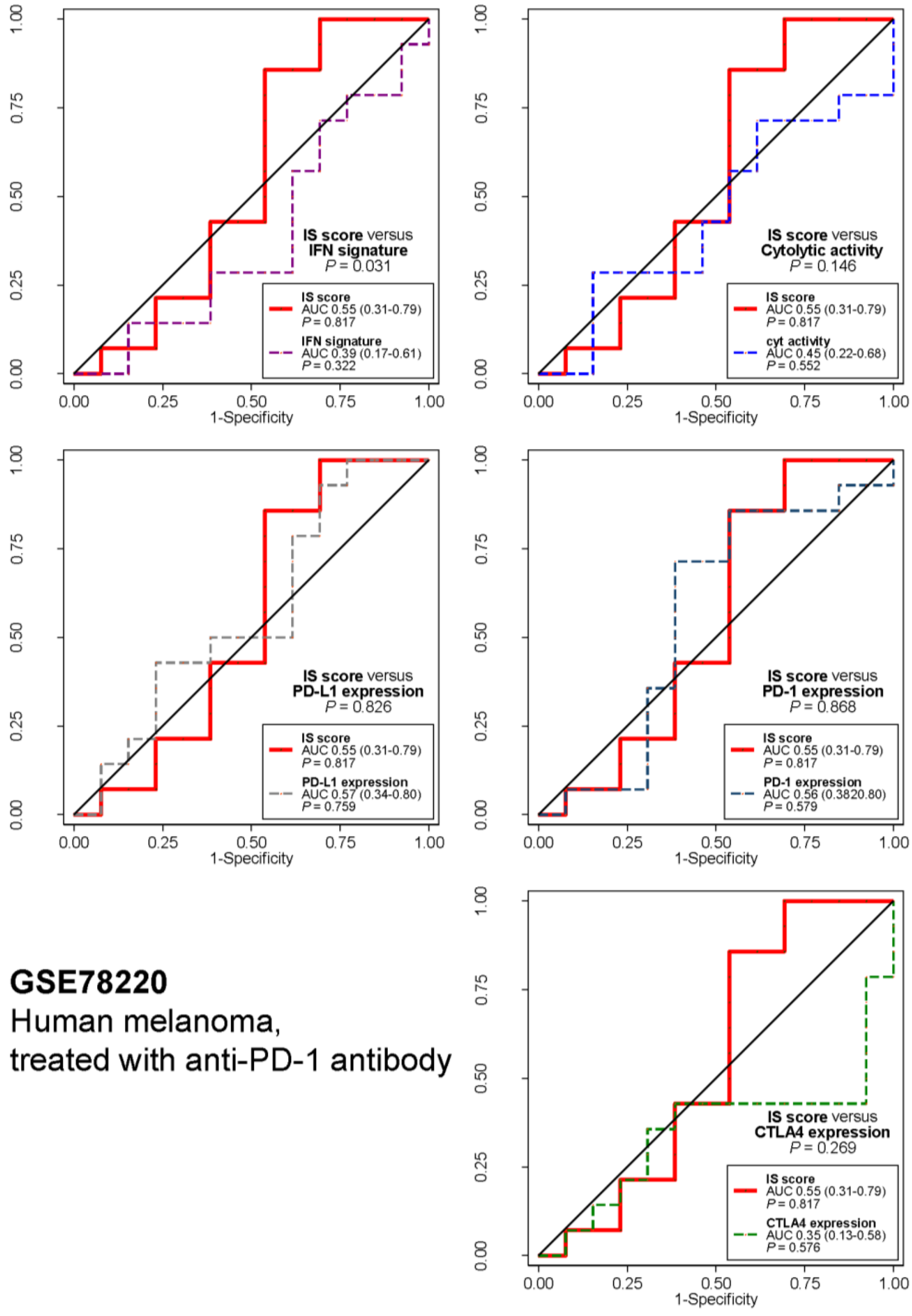

Supplementary Figure 4. Prognostic significance of immune signature (IS) score in human melanoma treated with anti-PD-1 antibody

Receiver operating characteristics (ROC) analysis of various immune biomarkers as well as IS scores from each prediction (top left, interferon-gamma (IFN) signature; top right, cytolytic activity; middle left, PD-L1 expression; middle right, PD-1 expression; bottom, CTLA-4 expression). Significance of IS biomarkers was estimated by area under curve (AUC) from ROC analysis.

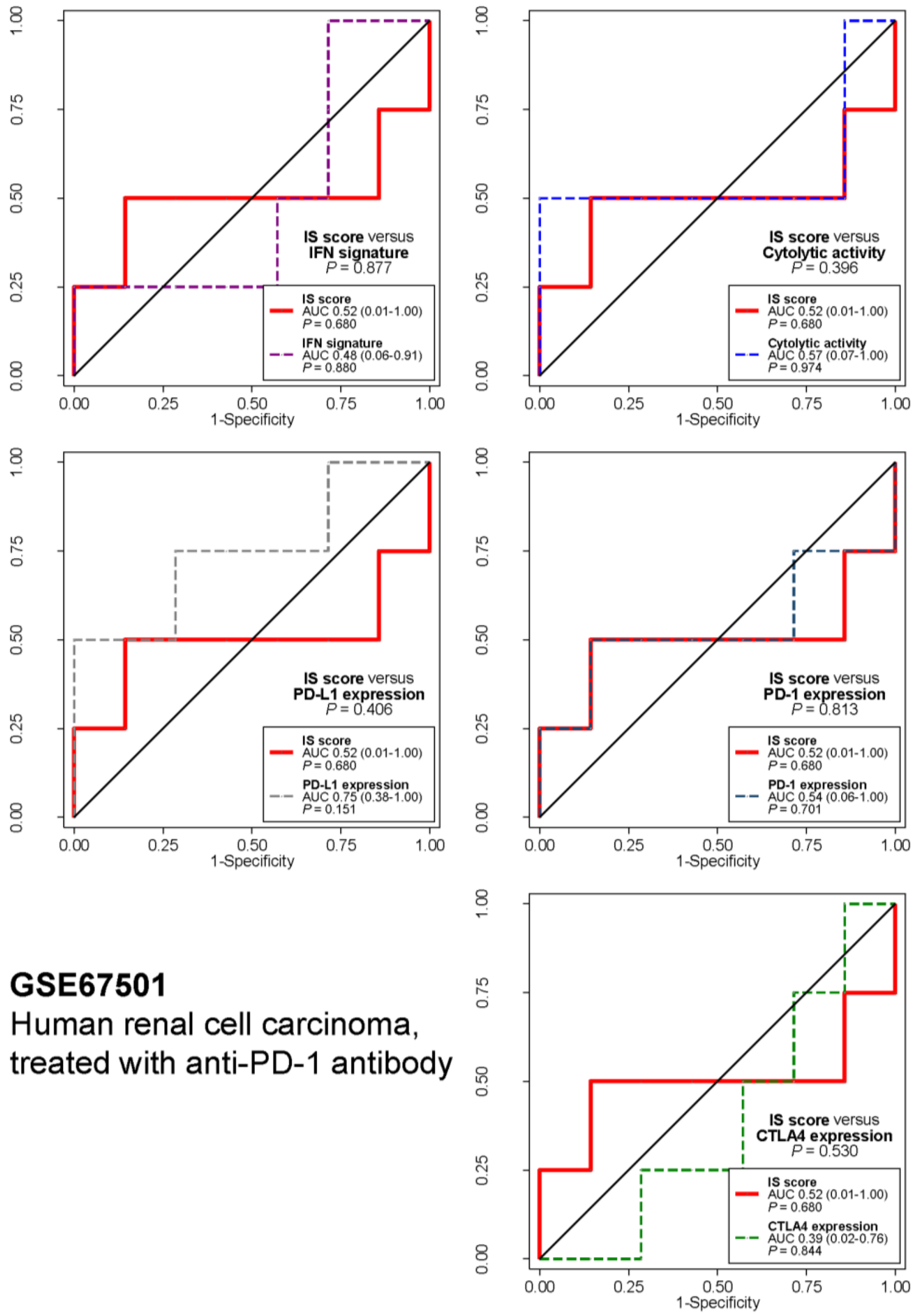

Supplementary Figure 5. Prognostic significance of immune signature (IS) score in human renal cell carcinoma treated with anti-PD-1 antibody

Receiver operating characteristics (ROC) analysis of various immune biomarkers as well as IS scores from each prediction (top left, interferon-gamma (IFN) signature; top right, cytolytic activity; middle left, PD-L1 expression; middle right, PD-1 expression; bottom, CTLA-4 expression). Significance of IS biomarkers was estimated by area under curve (AUC) from ROC analysis.

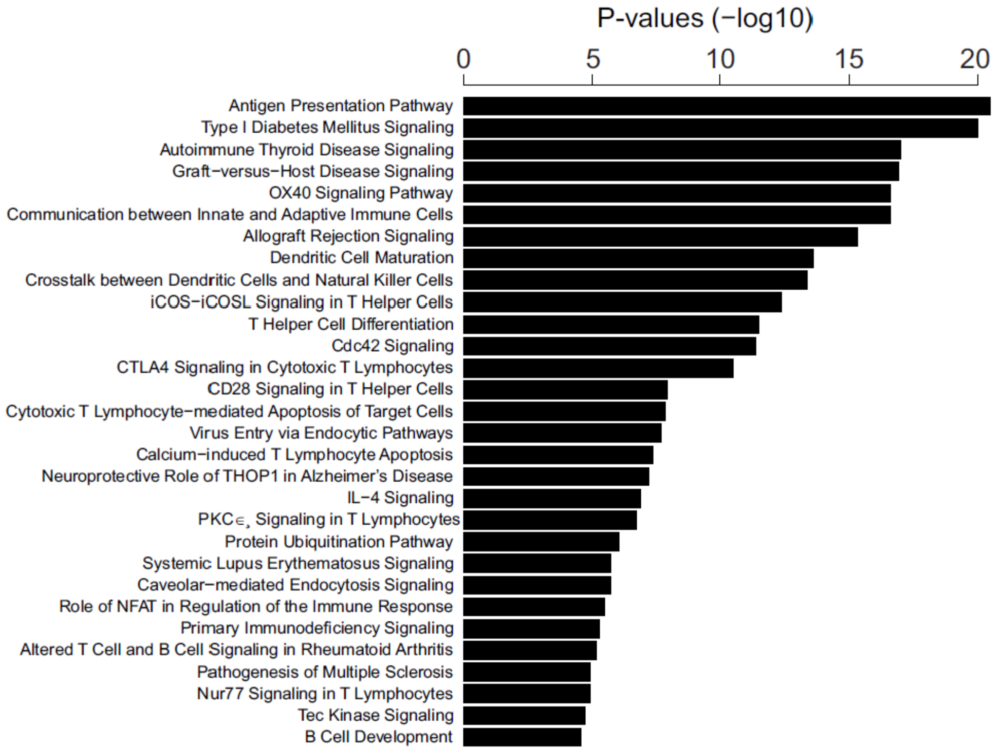

Supplementary Figure 6. Canonical signaling pathways enriched in immune signature genes
Immune signature genes identified in Figure 1 were analyzed by IPA™ for enrichment of canonical pathways activated in responders to immunotherapy. Bars indicate −log P values that were generated by Fisher's exact test.

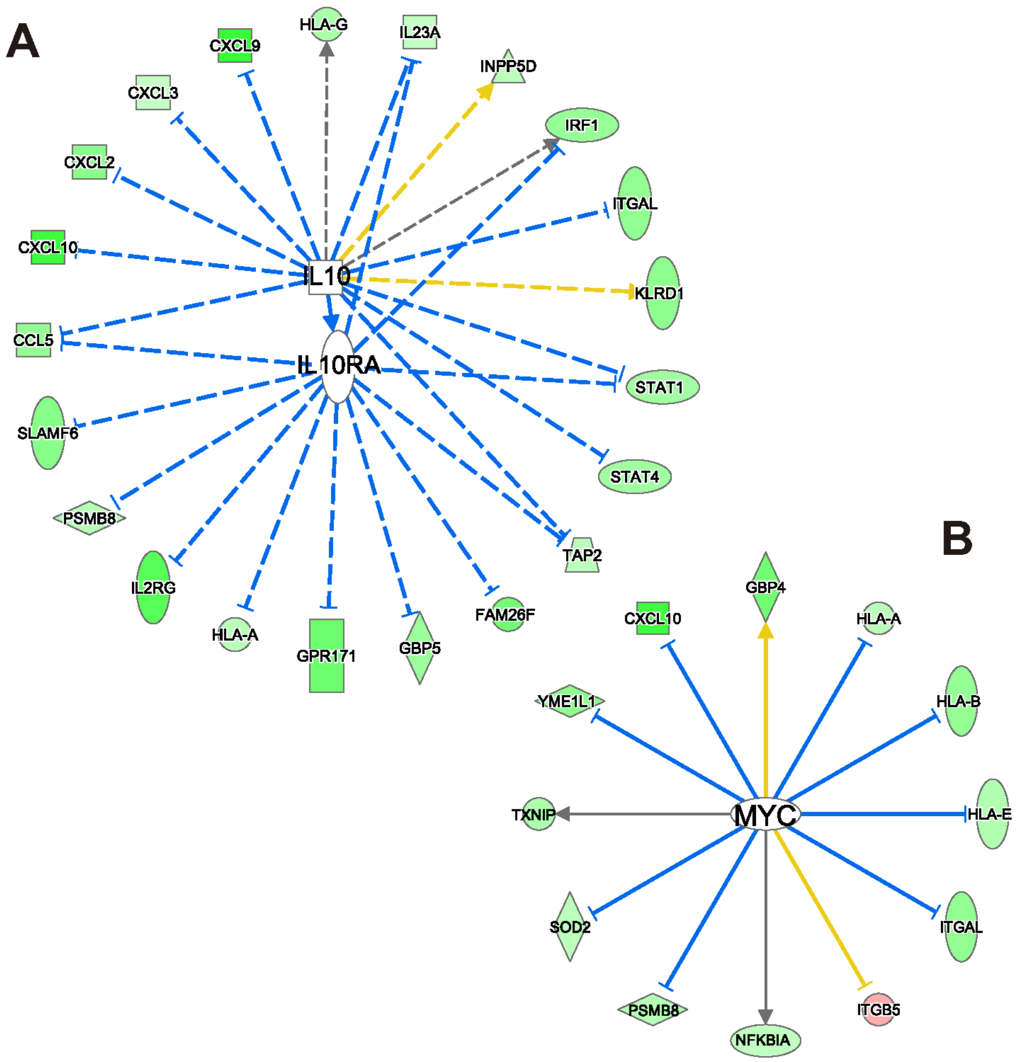

Supplementary Figure 7. Gene network analysis with genes down-regulated in non-responder patients

Analysis using Ingenuity Pathway Analysis software revealed that networks of immune genes repressed by *IL10*, *IL10RA* (A), and *MYC* (B) were significantly down-regulated in non-responders, indicating that these genes are activated in non-responders. Upregulated and downregulated genes in non-responders are indicated by red and green, respectively. The lines and arrows represent functional and physical interactions and directions of regulation from the literature.

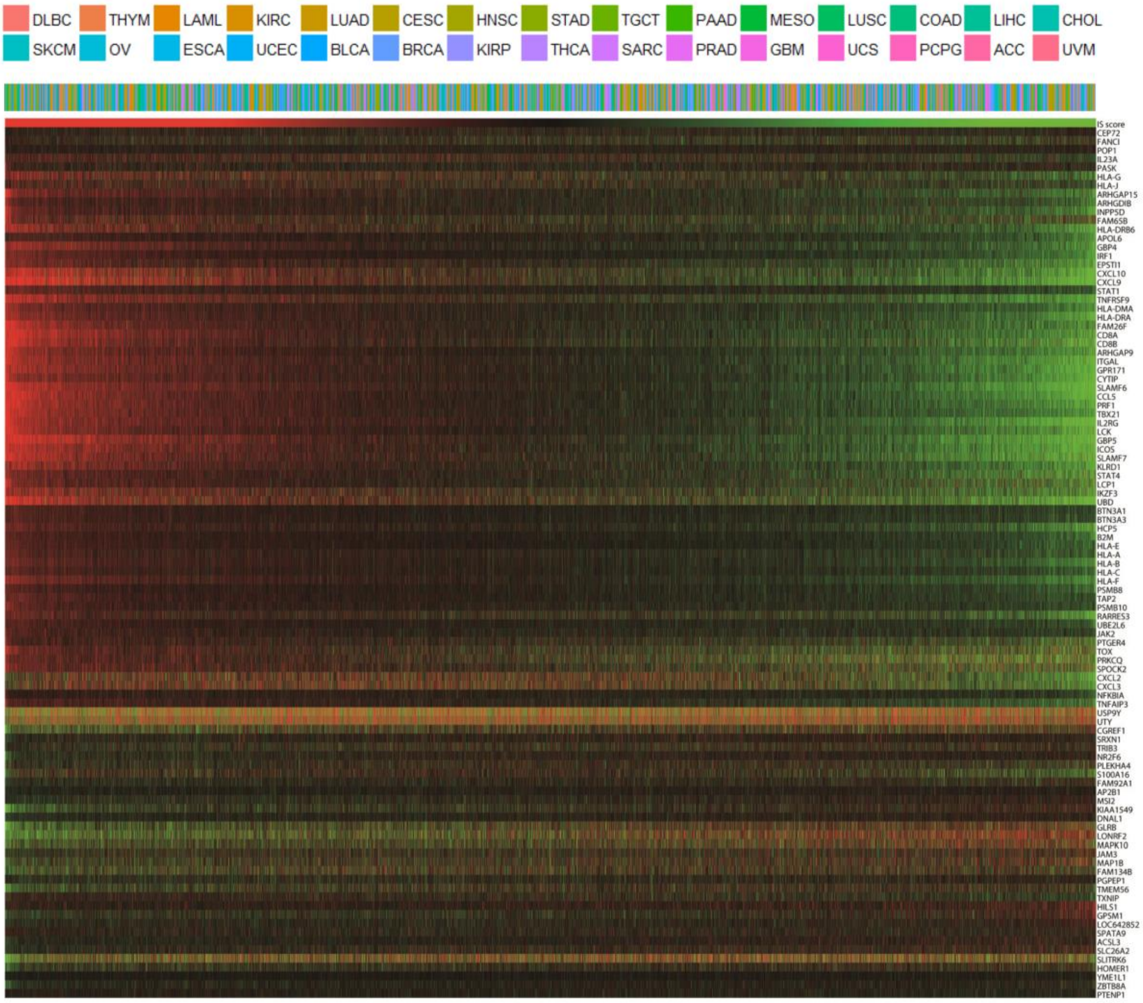

Supplementary Figure 8. Immune signature gene expressions of The Cancer Genome Atlas (TCGA) tumor samples

Heatmap of gene expression included in immune signature (IS) score was plotted (red, high expression; green, low expression). Abbreviation of cancer type was referred from The Cancer Genome Atlas tag name.

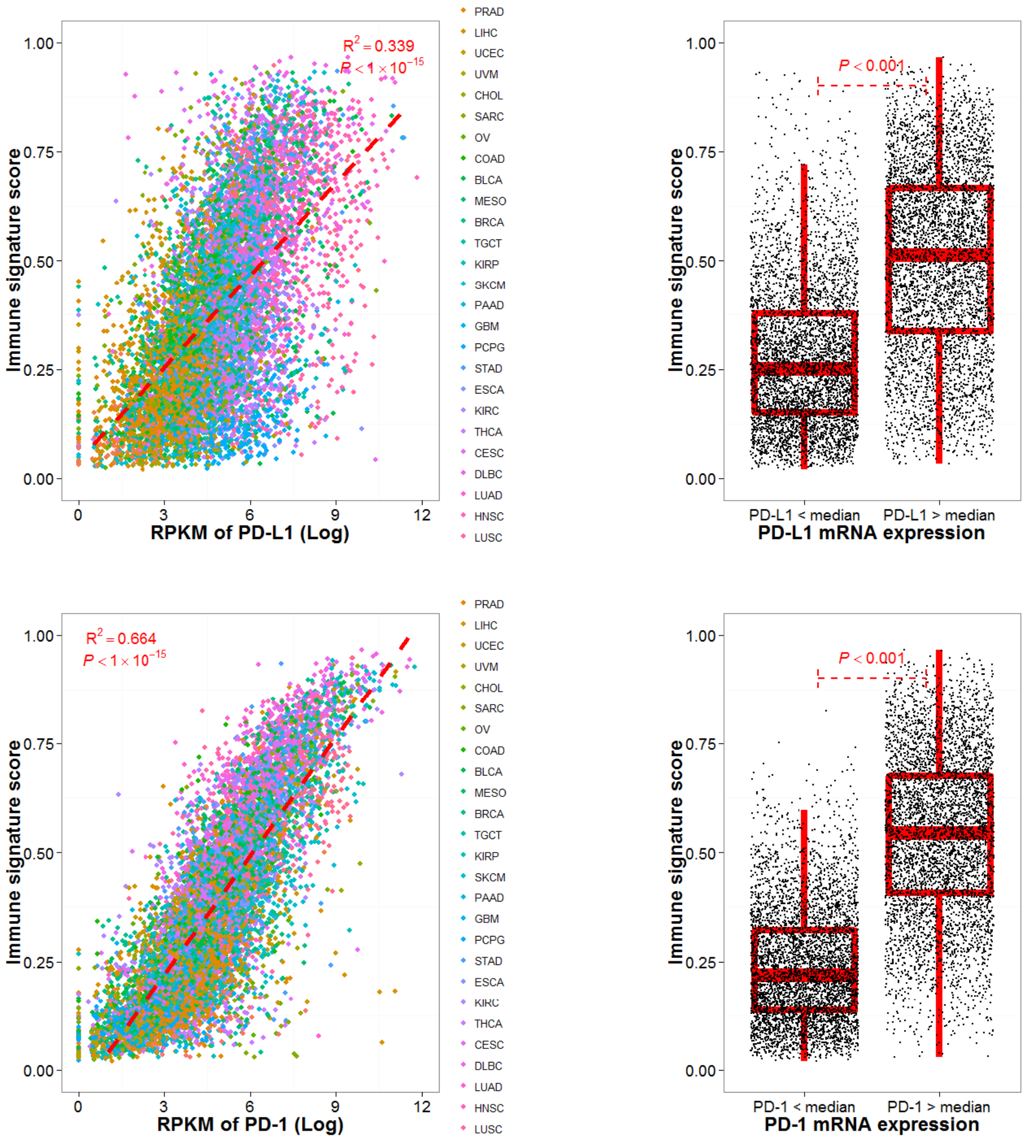

Supplementary Figure 9. Association of PD-L1 and PD-1 expressions with immune signature scores

Scatter plot (left) and box plot (right) of IS score and reads per kilobase of transcript per million reads mapped (RPKM) of programmed death-ligand1 (PD-L1, top), and programmed death-1 (PD-1, bottom) across cancer types. Abbreviation of cancer type was referred from The Cancer Genome Atlas tag name; n/a, not applicable.

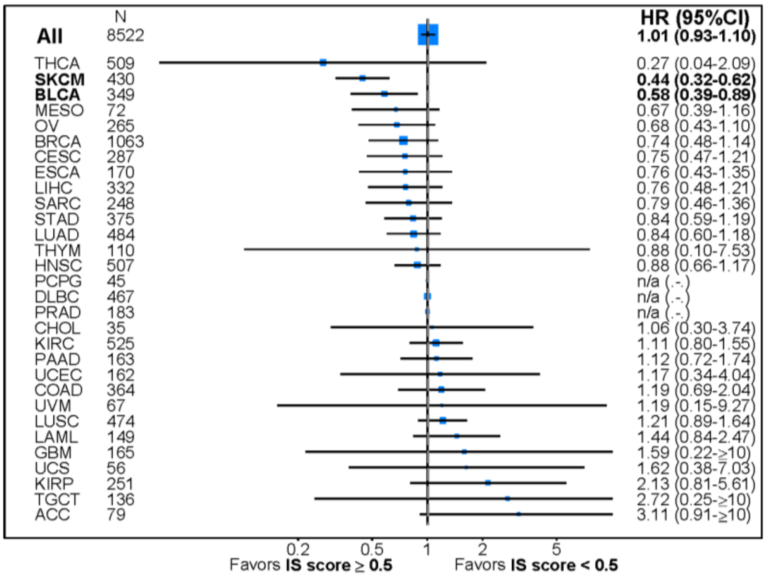

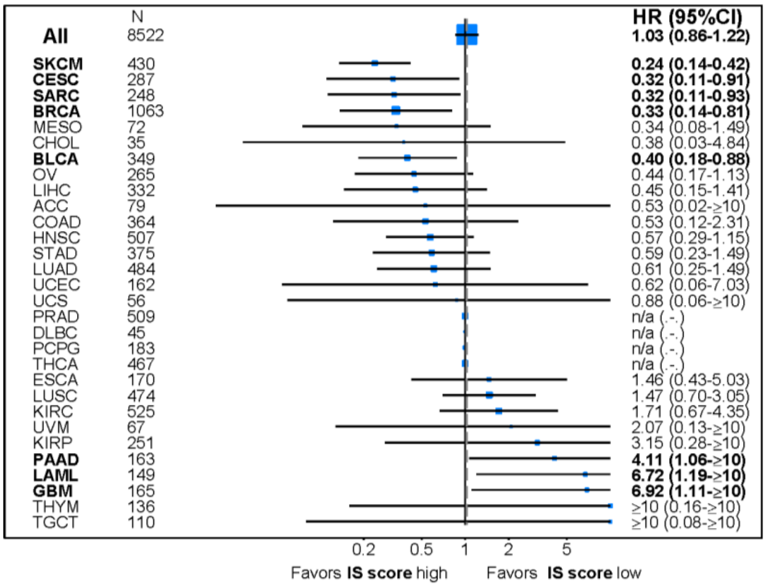

Supplementary Figure 10. Subgroup analysis of overall survival according to IS score
Forest plot of hazard ratio (HR) and 95% confidence interval (CI) by Cox regression survival analysis according to two groups dichotomized by immune signature score (top) and continuous value of IS score (bottom) across cancer types. Abbreviation of cancer type was referred from The Cancer Genome Atlas tag name; n/a, not applicable.

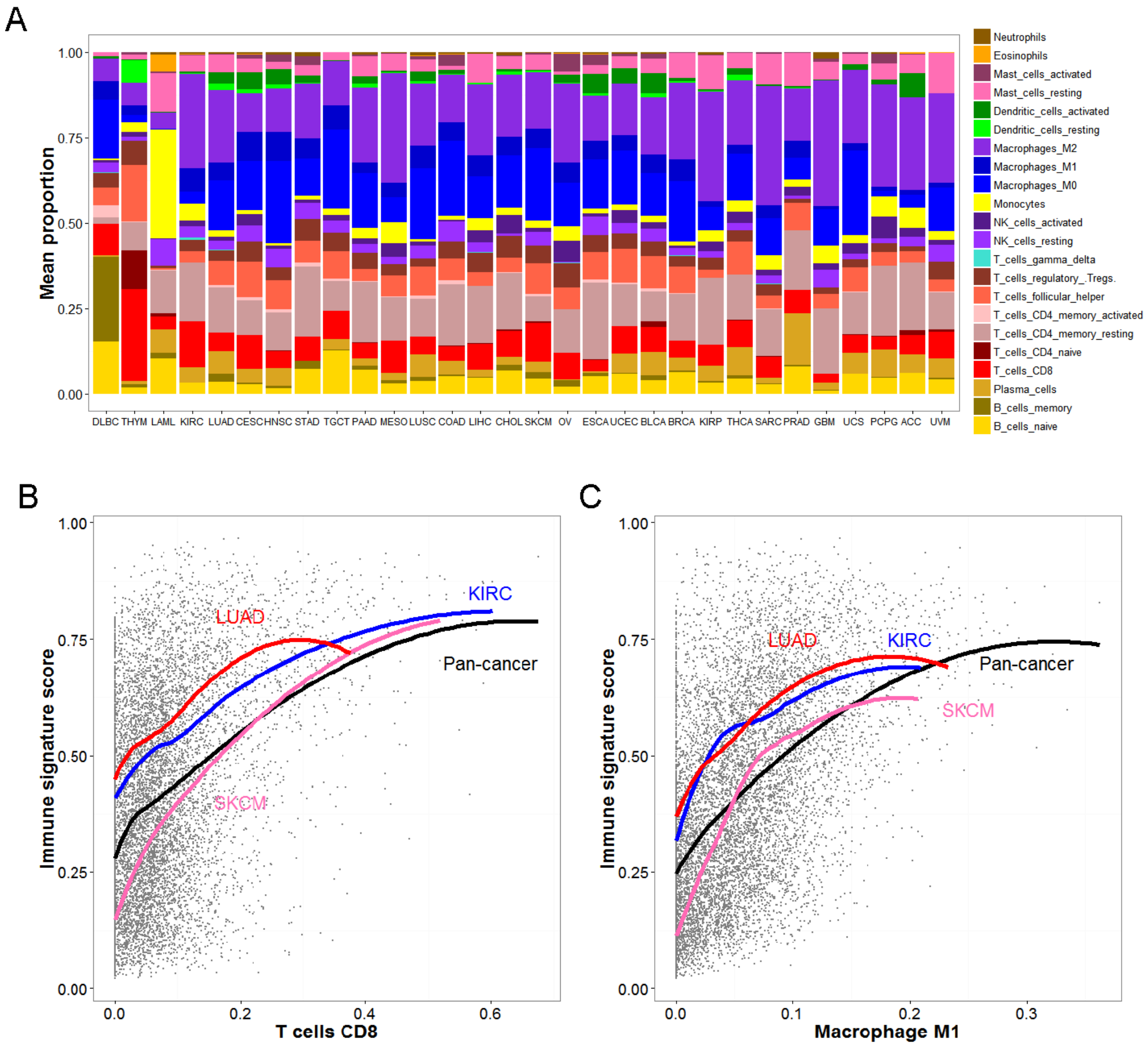

Supplementary Figure 11. Association of Immune signature score and immune cell properties calculated by Cibersort[1]

Immune cell proportions in each cancer type (A). Each bar representatives mean proportion of immune cell types calculated by Cibersort algorism. Scatter plot of IS score and CD8 T cell (B) or Macrophage M1 (C) proportions. Solid lines represent local regression curves between IS score and immune cell proportions in each cancer as indicated. Abbreviation: LUAD, lung adenocarcinoma; KIRC, renal clear cell carcinoma; SKCM, skin cutaneous melanoma.

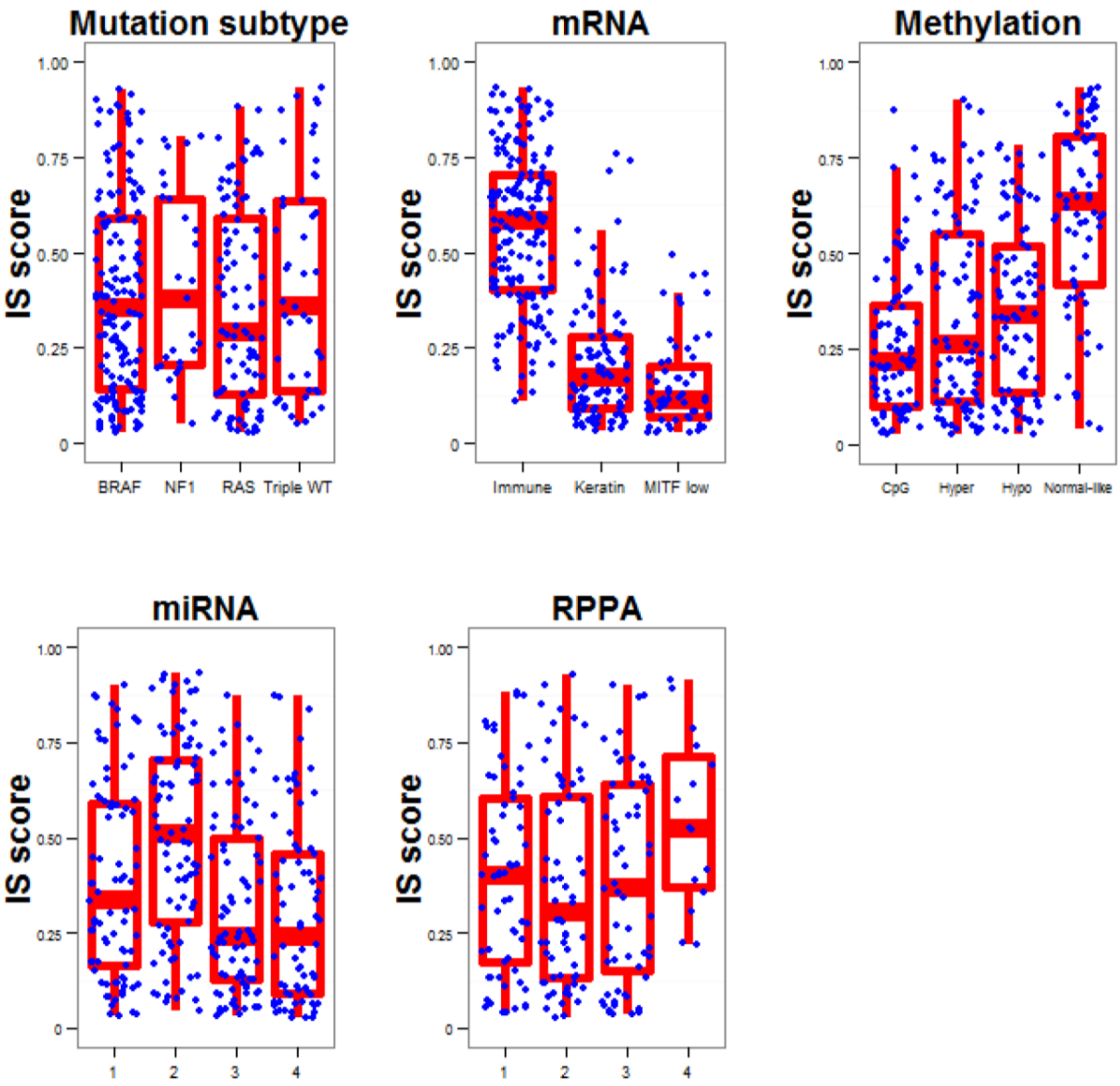

Supplementary Figure 12. Potential response of patients with melanoma to immunotherapy
Tumors were grouped according to molecular subtypes that were discovered by TCGA study on melanoma (SKCM).

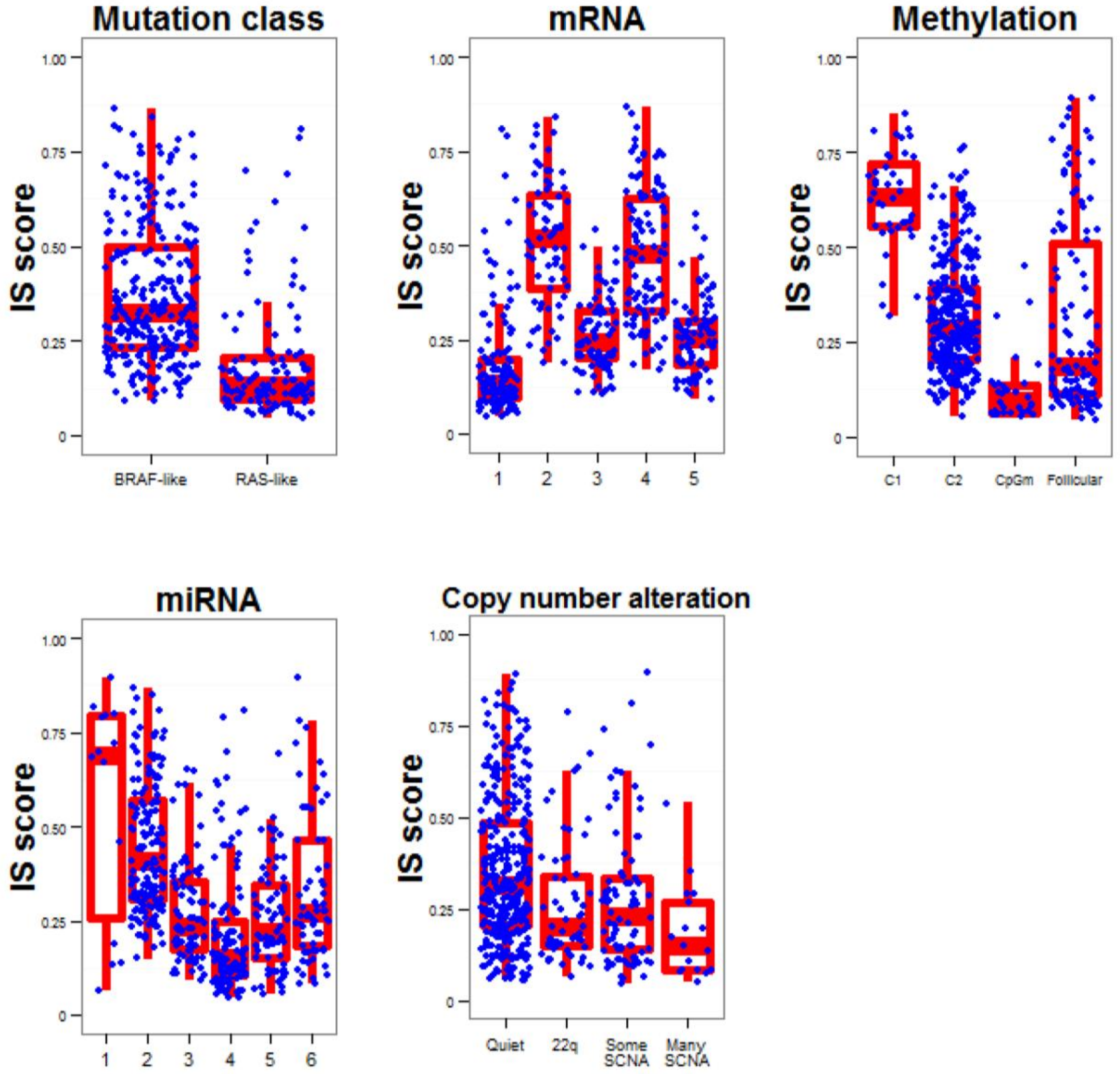

Supplementary Figure 13. Potential response of patients with thyroid cancer to immunotherapy
Tumors were grouped according to molecular subtypes that were discovered by TCGA study on thyroid cancer (THCA).

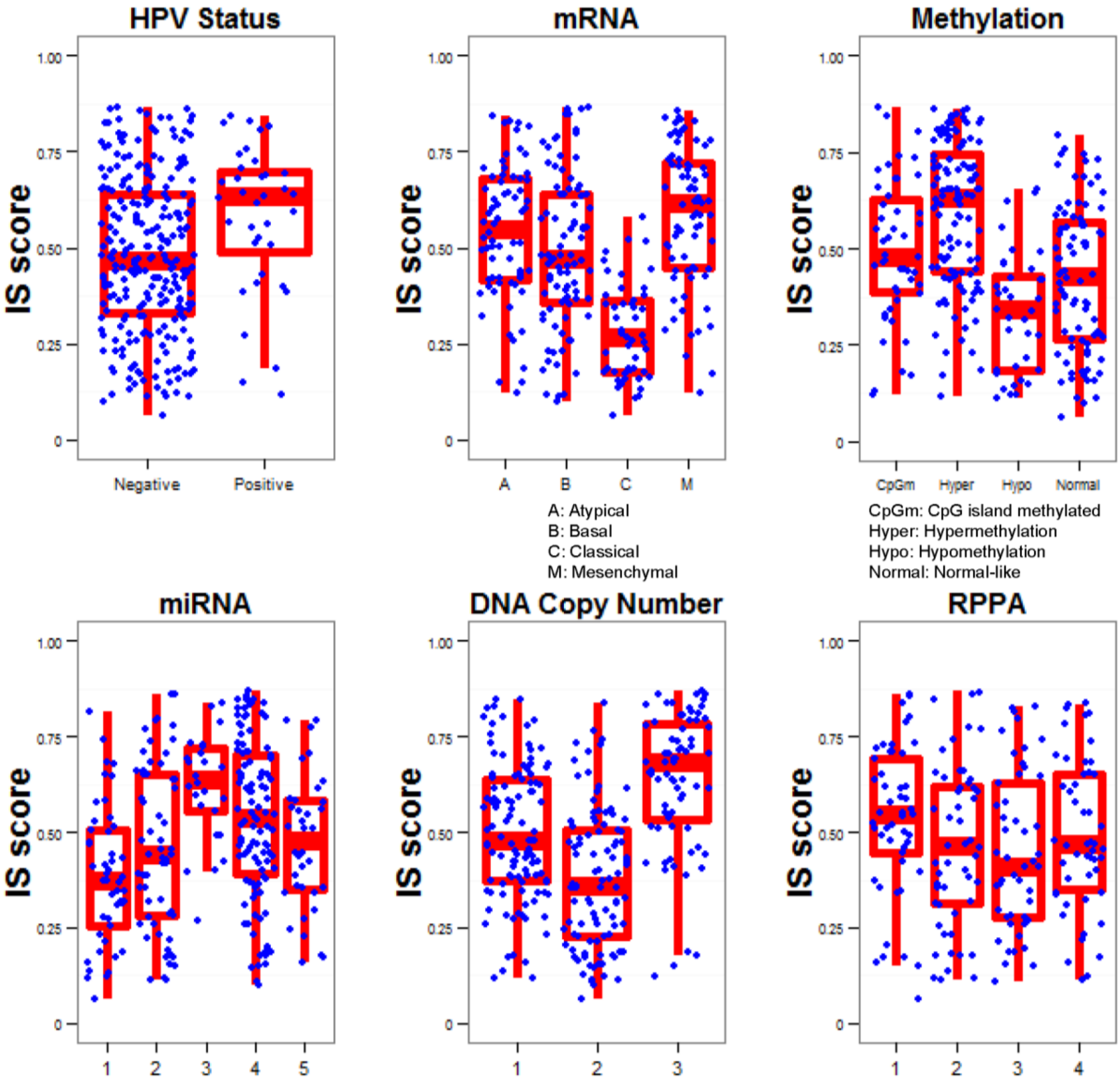

Supplementary Figure 14. Potential response of patients with head and neck squamous cell carcinoma to immunotherapy
Tumors were grouped according to molecular subtypes that were discovered by TCGA study on head and neck squamous cell carcinoma (HNSC).

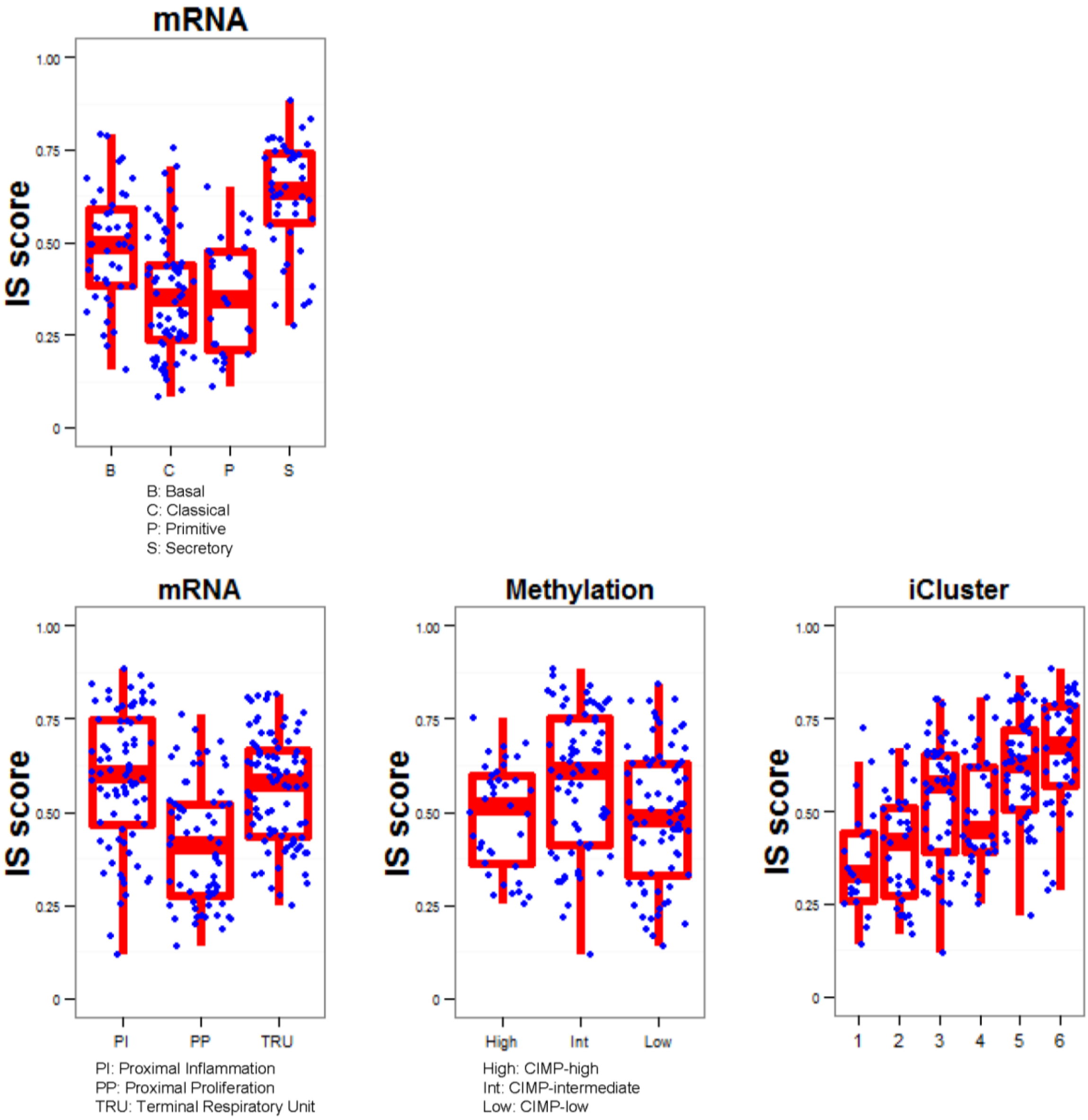

Supplementary Figure 15. Association of molecular subtypes of HNSC with IS scores
(top) Patients were stratified according to HPV status first and platform-based molecular subtypes subsequently as indicated.
(bottom) Membership of tumors in molecular subtypes associated with IS scores.

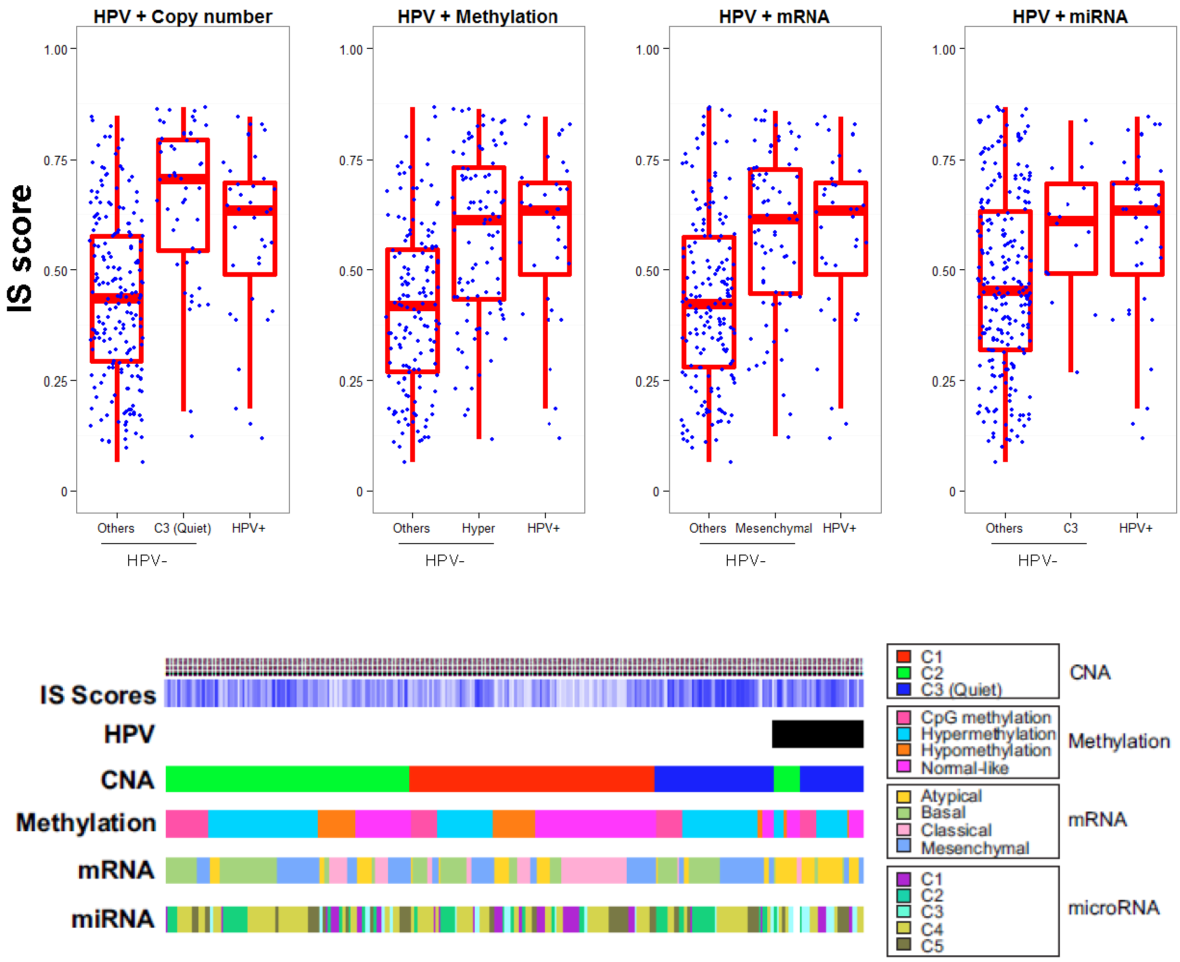

Supplementary Figure 16. Potential response of patients with lung cancer to immunotherapy (top) Lung squamous cell carcinoma (LUSC) were grouped by mRNA molecular subtypes. (bottom) Lung adenocarcinoma (LUAD) were grouped by molecular subtypes.

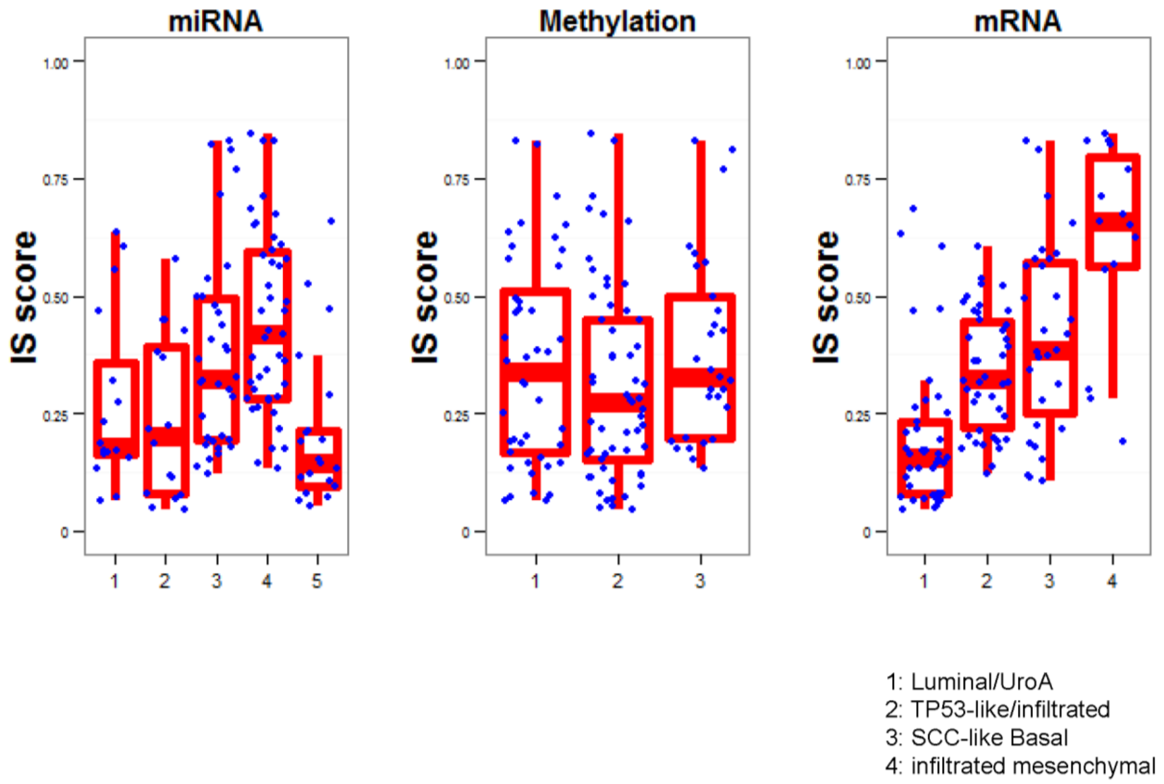

Supplementary Figure 17. Potential response of patients with muscle-invasive bladder cancer to immunotherapy

Tumors were grouped according to molecular subtypes that were discovered by TCGA study on muscle-invasive bladder cancer (BLCA).

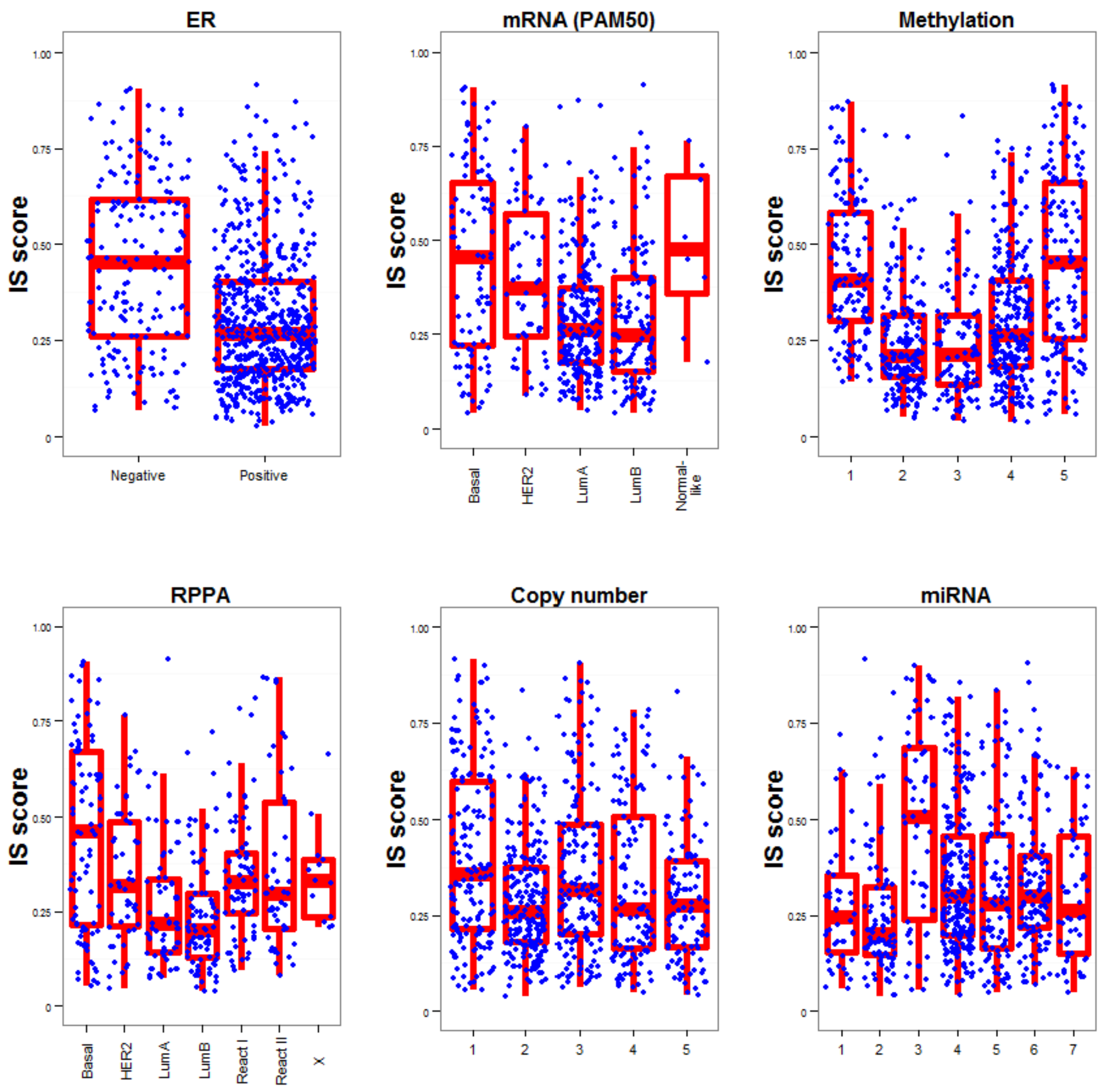

Supplementary Figure 18. Potential response of patients with breast cancer to immunotherapy
Tumors were grouped according to molecular subtypes that were discovered by TCGA study on breast cancer (BRCA).

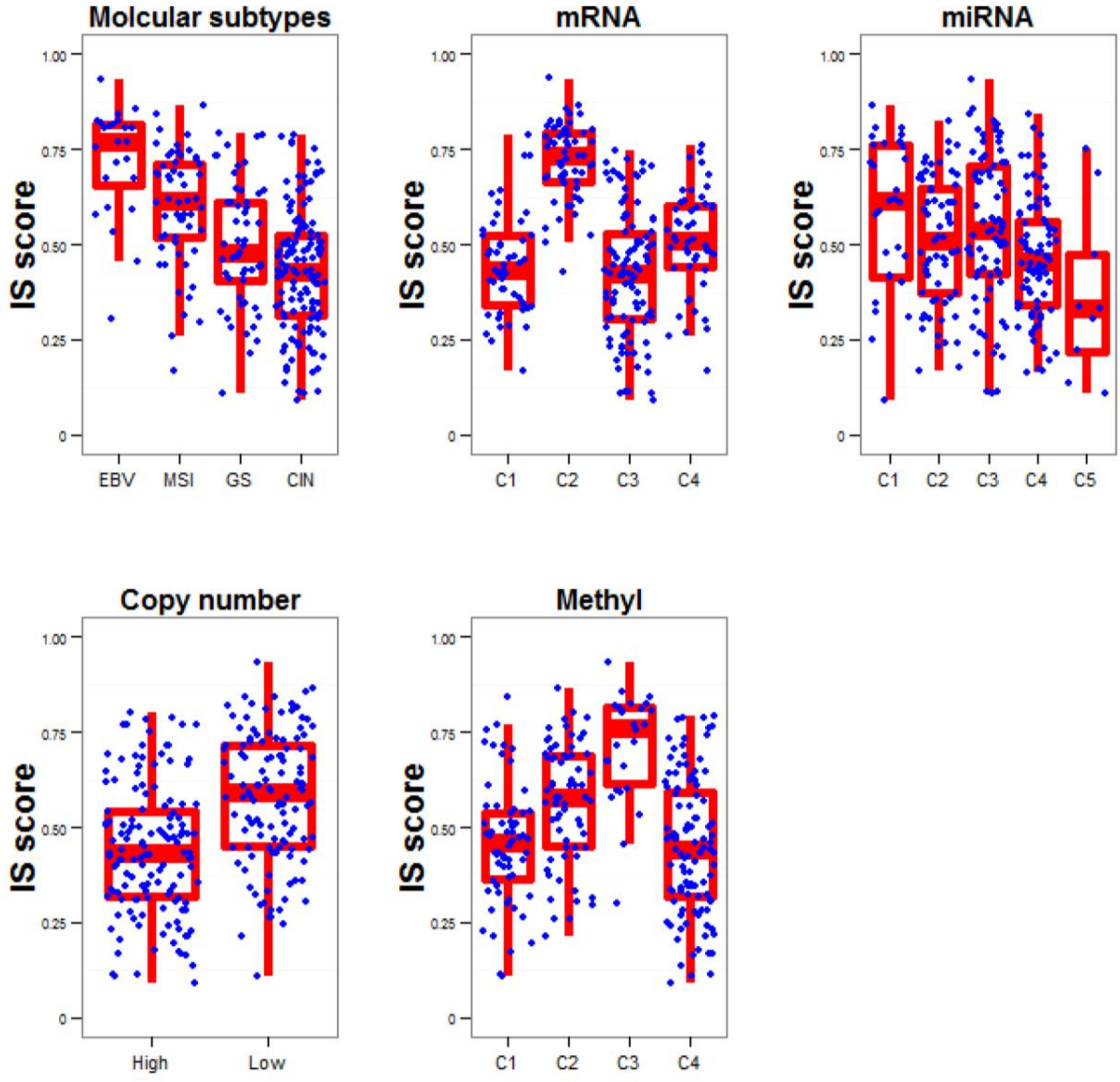

Supplementary Figure 19. Potential response of patients with stomach cancer to immunotherapy
Tumors were grouped according to molecular subtypes that were discovered by TCGA study on stomach cancer (STAD).

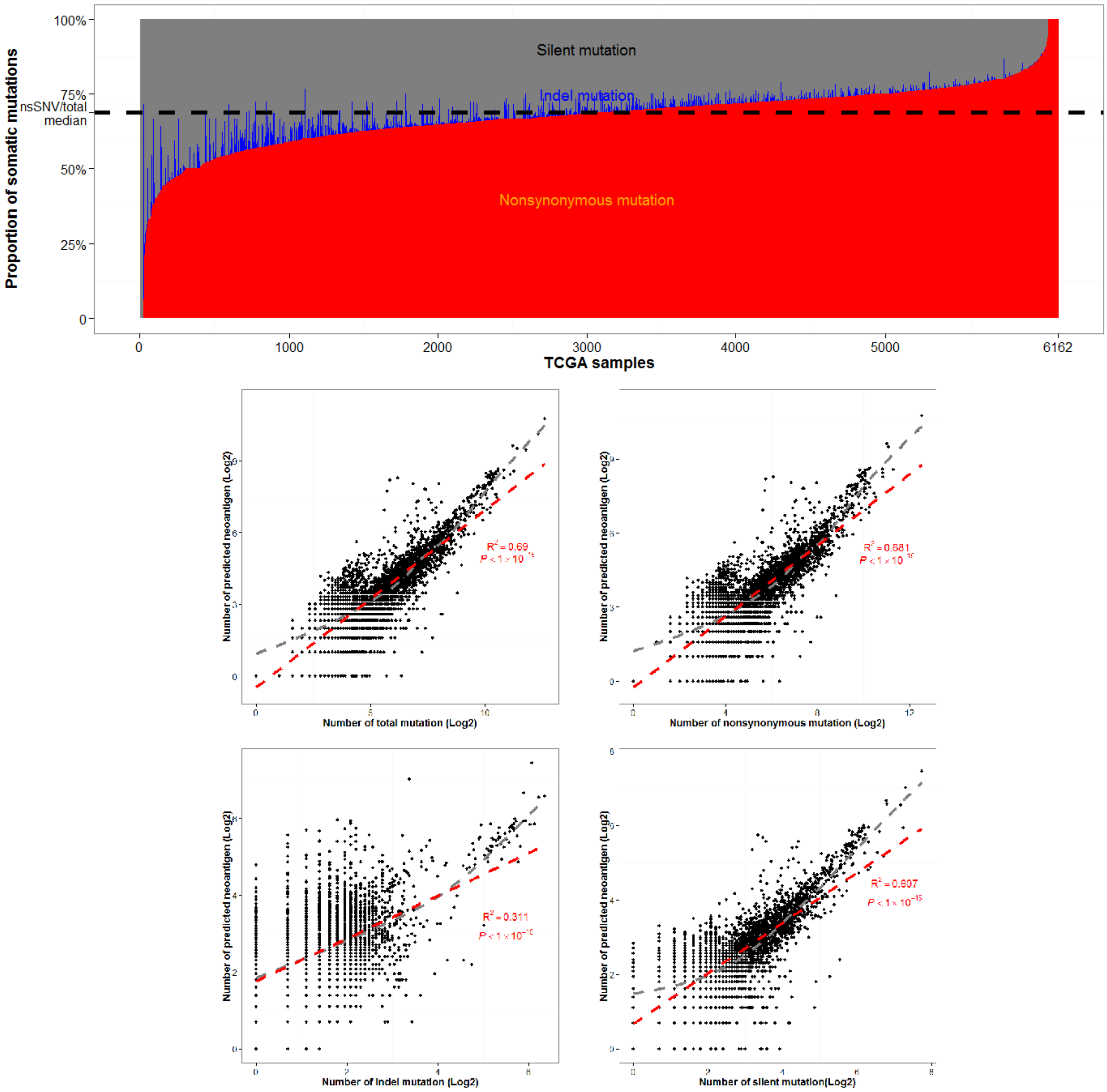

Supplementary Figure 20. Landscape of somatic mutation in TCGA samples.
(top) Percentage of nonsynonymous (red), indel (blue), silent (gray) mutations in tissue were plotted. (bottom) The number of total mutation (nonsynnymous + indel + silent, upper left), nonsynonymous (upper right), indel (lower left), and silent mutation (lower right) were compared with predicted neoantigen from the previous study[2].

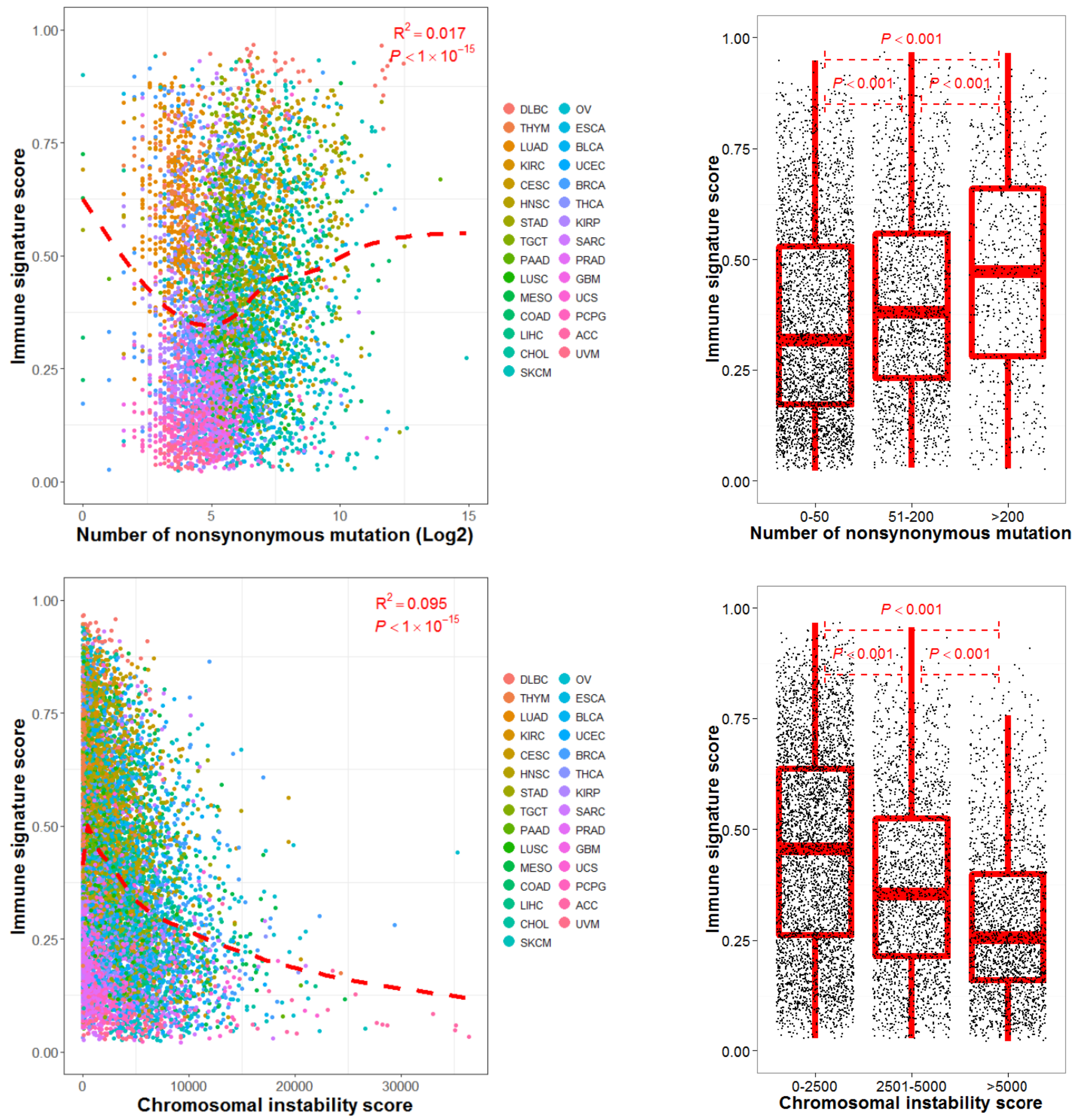

Supplementary Figure 21. Significant correlation of immune signature with mutational load and copy number alteration.

(top) Scatter plot (left) and box plot (right) of immune signature (IS) score and number of mutation across cancer types.

(bottom) Scatter plot (left) and box plot (right) of IS score and chromosomal instability score.

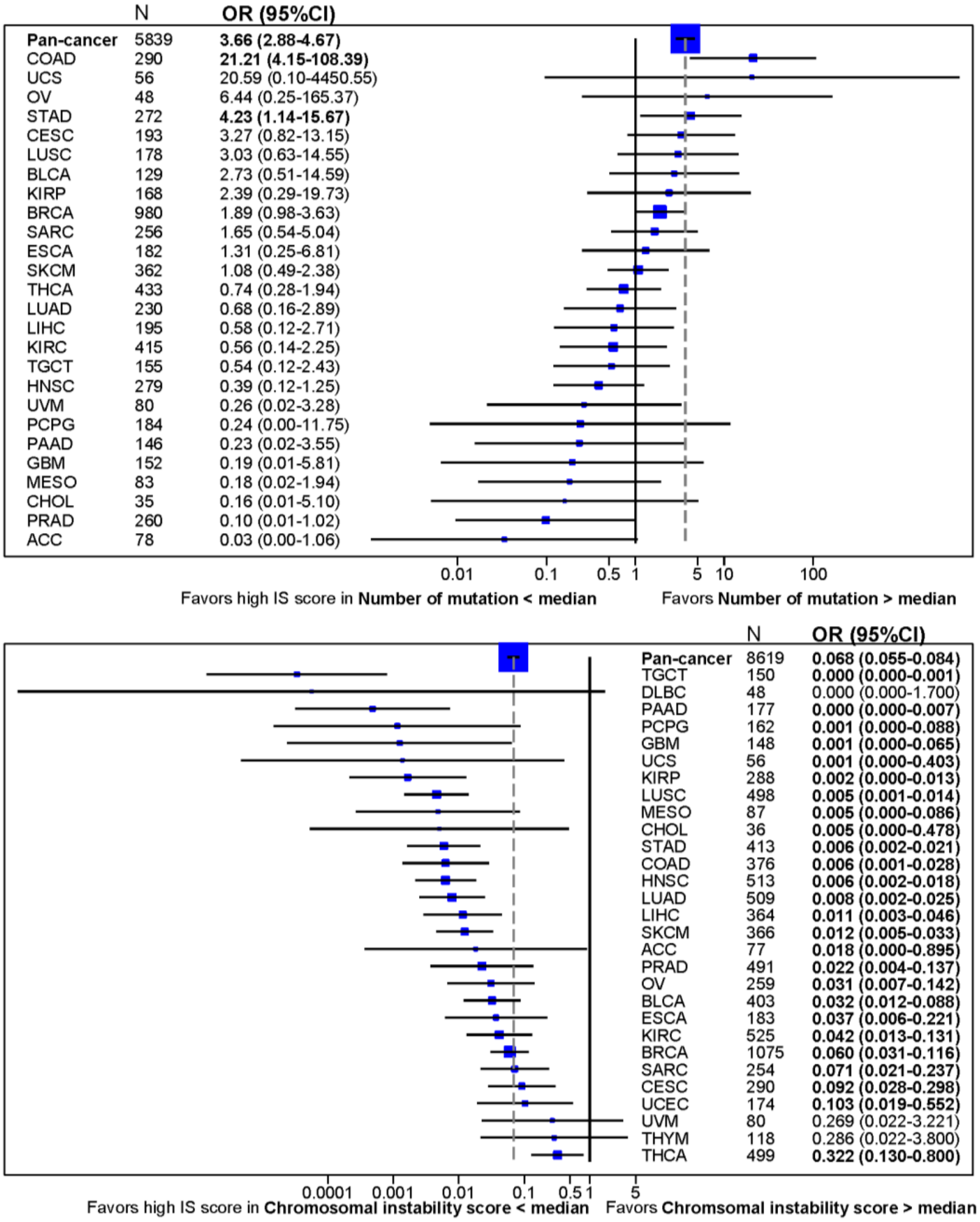

Supplementary Figure 22. Forest plot of odds ratio (OR) and 95% confidence interval (CI) by logistic regression analysis of association of IS scores with number of mutation (top) and chromosomal instability score (bottom).

Tumors were dichotomized by median of number of mutations or CIN scores.

Abbreviation of cancer type was referred from The Cancer Genome Atlas tag name; IS, immune signature.

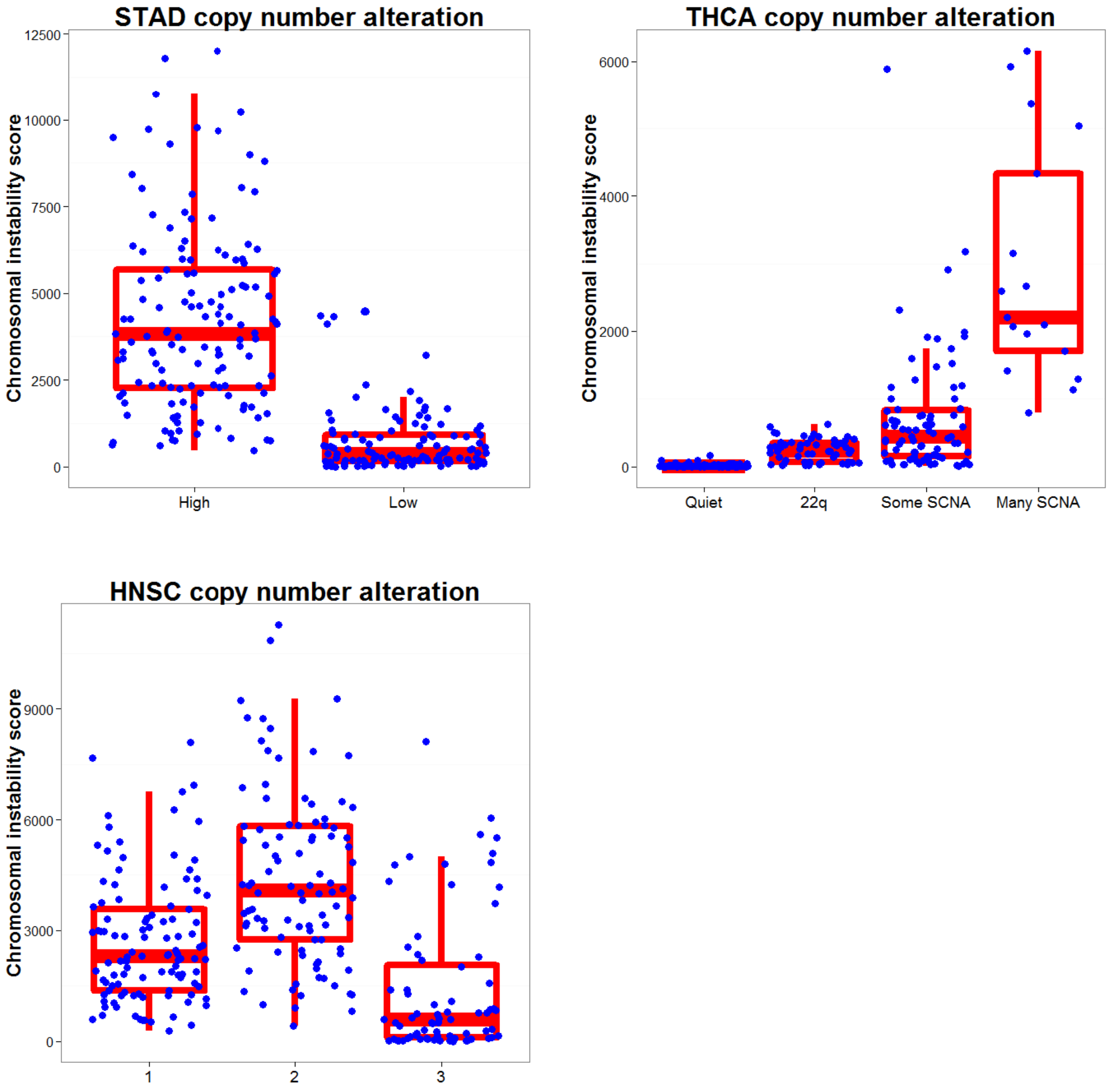

Supplementary Figure 23. Validation of chromosomal instability score.
Chromosomal instability (CIN) score according to previously reported copy number alteration cluster of stomach adenocarcinoma (STAD, top left), thyroid cancer (THCA, top right), and head and neck cancer (HNSC, bottom left) were compared.

Supplementary Figure 6E

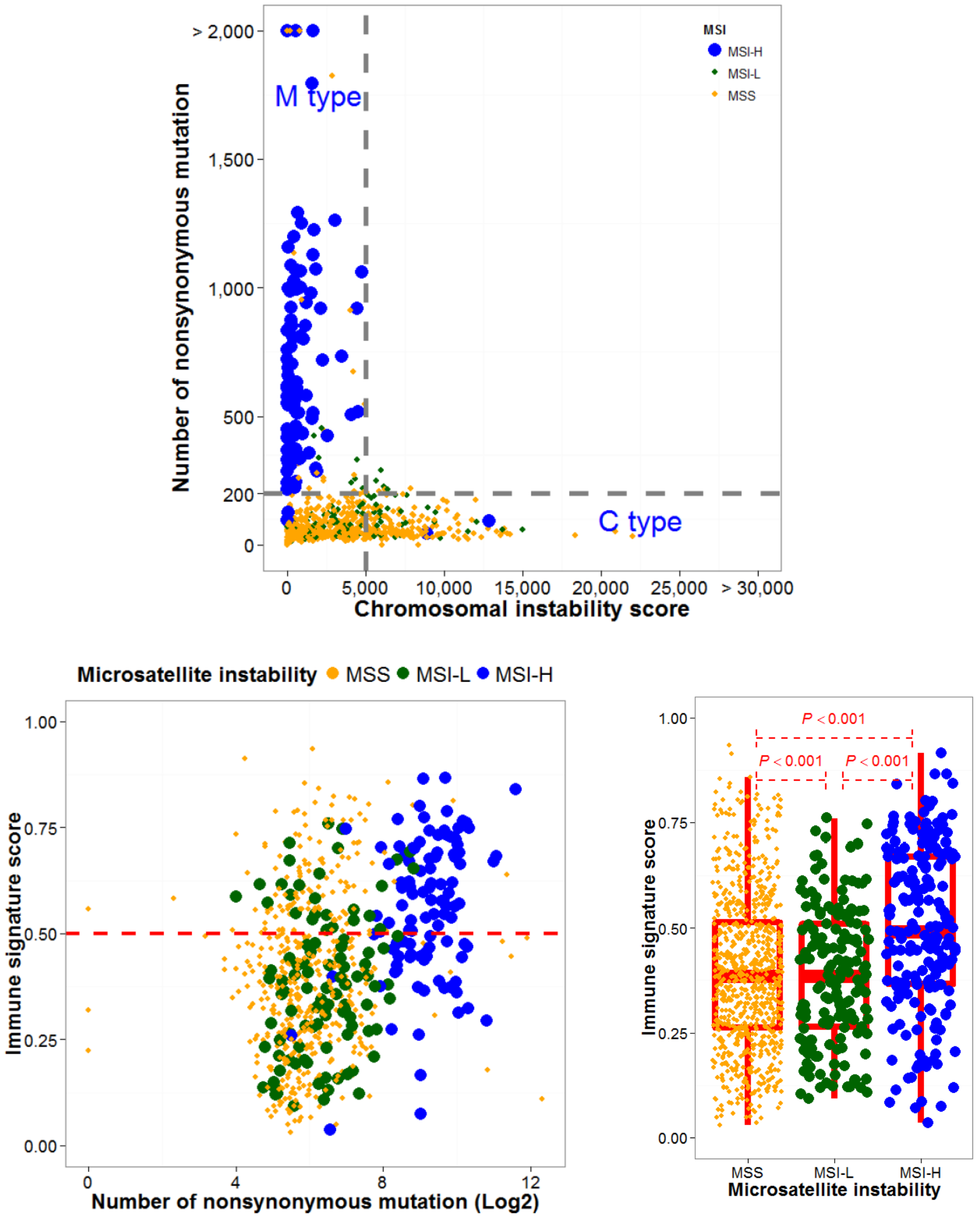

Supplementary Figure 24. Significant correlation of immune signature with microsatellite status. Scatter plot (top) and box plot (bottom) of immune signature score and number of mutation according to microsatellite (MSI)-status.

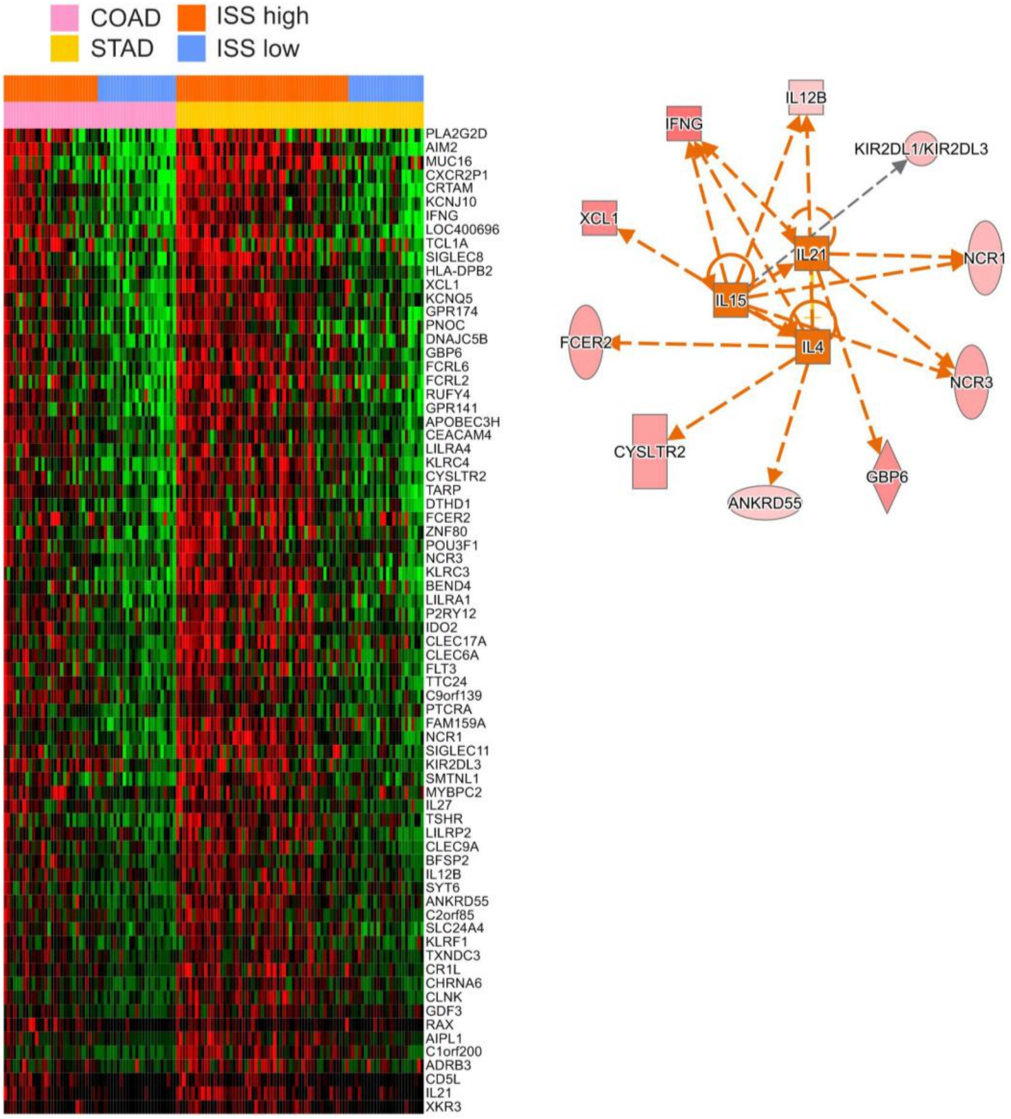

Supplementary Figure 25. Over-expressed genes in tumors with high IS scores in MSI-H colorectal cancer and stomach cancer.
(Left) Heatmap of genes differentially expressed between tumors with high or low IS scores in MSI-H colorectal cancer (COAD) and stomach cancer (STAD). Seventy two were identified as commonly up-regulated genes ($P < 0.005$) in high IS score tumors in both COAD and STAD data.
(Right) Gene network enriched in tumors with high IS scores. Analysis using Ingenuity Pathway Analysis software revealed that networks of genes regulated by IL4, IL21, and IL15 were significantly up-regulated in IS score high tumors. Upregulated and downregulated genes in non-responders are indicated by red and green, respectively. The lines and arrows represent functional and physical interactions and directions of regulation from the literature.

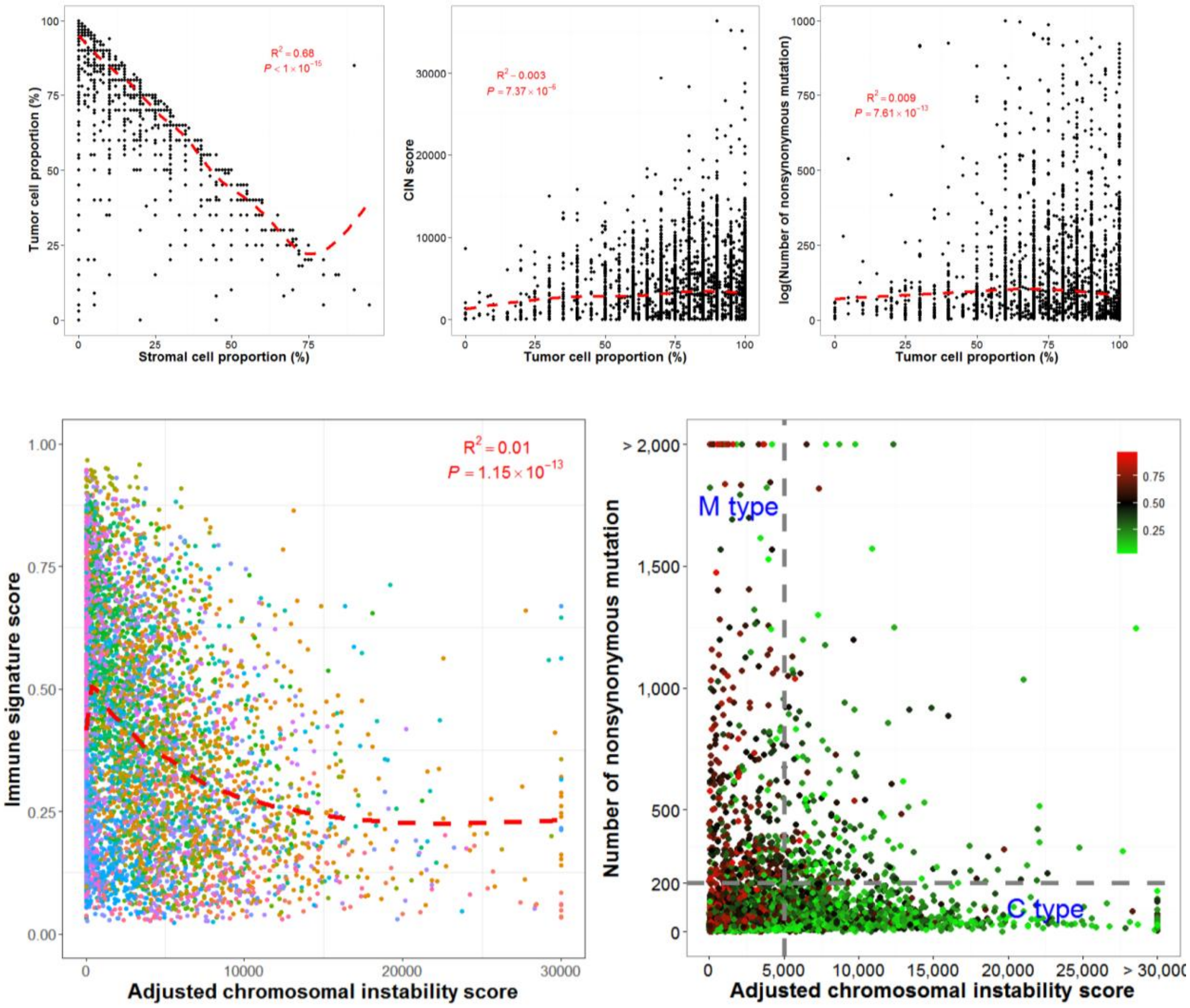

Supplementary Figure 26. Chromosomal instability score according to the tumor purity in TCGA samples.

(top) Scatter plot of the proportion of tumor cell and stromal cell (left), chromosomal instability (CIN) score and tumor cell proportion (middle), and number of nonsynonymous mutation and tumor cell proportion were plotted.

(bottom) Scatter plot of IS score and adjusted chromosomal instability score (left). Scatter plot of number of nonsynonymous mutation (y axis) and adjusted CIN score (x axis) according to the degree of immune signature (IS) score (right).

Adjusted CIN score = CIN score / tumor purity.

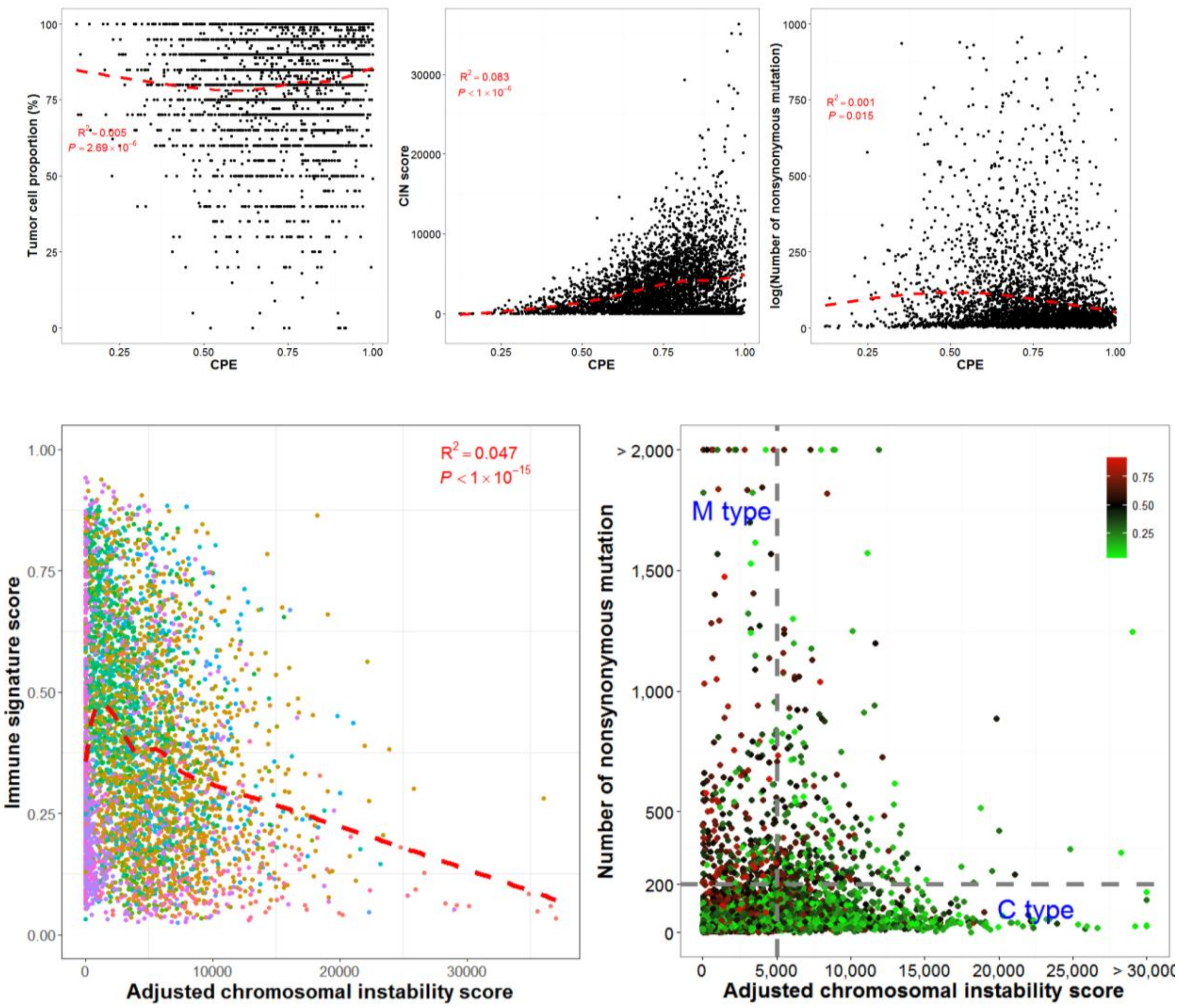

Supplementary Figure 27. Adjusted chromosomal instability score according to the tumor purity in TCGA samples.

(top) Scatter plot of the proportion of tumor cell and consensus measurement of purity estimations (CPE)[3] (left), chromosomal instability (CIN) score and CPE (middle), and number of nonsynonymous mutation and CPE (right) were plotted.

(bottom) Scatter plot of IS score and adjusted chromosomal instability score (left). Scatter plot of number of nonsynonymous mutation (y axis) and adjusted CIN score (x axis) according to the degree of immune signature (IS) score.

Adjusted CIN score = CIN score / CPE.

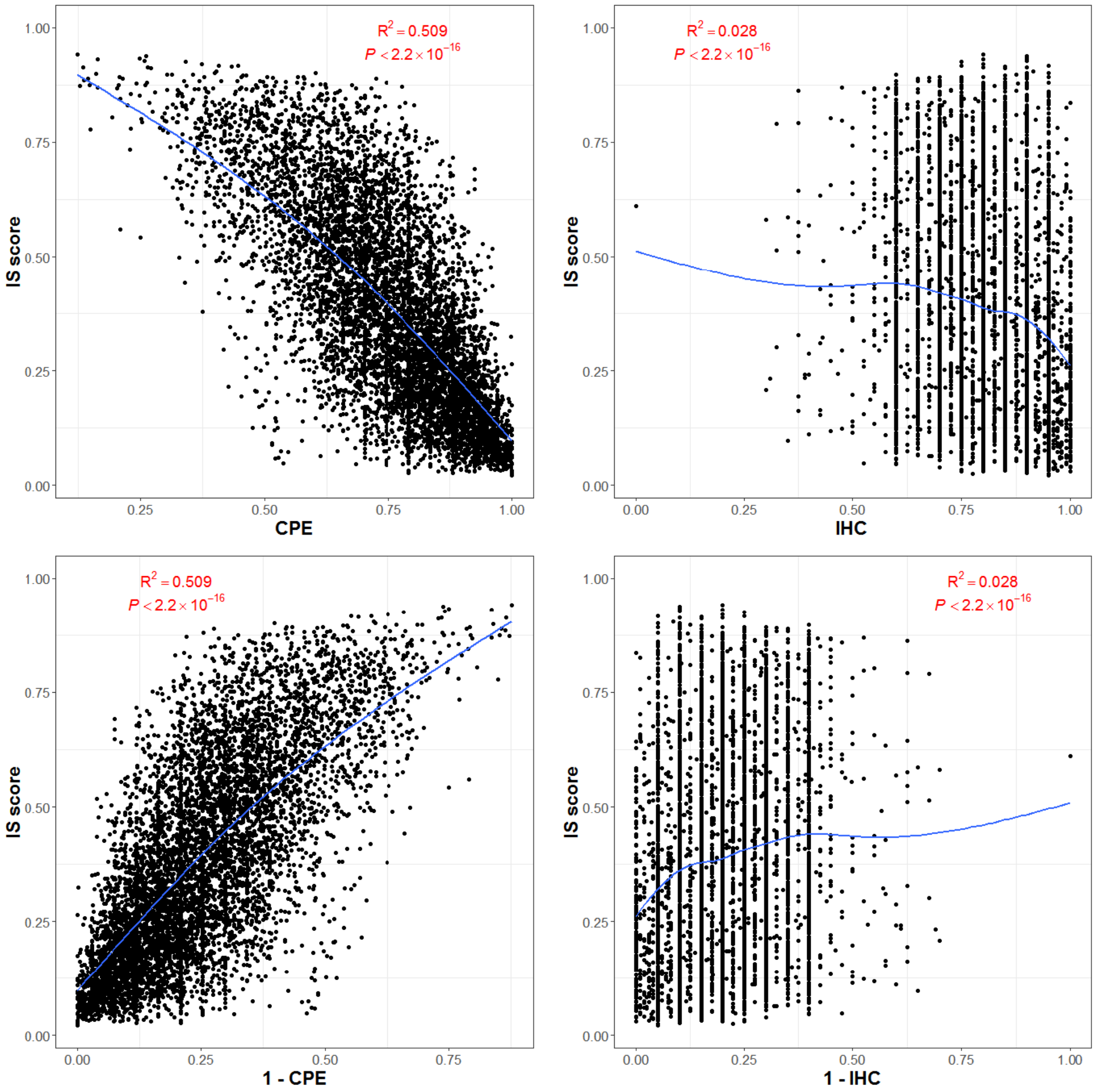

Supplementary Figure 28. Association of IS score and tumor purity in TCGA samples
(top) Scatter plot of immune signature score and the proportion of tumor cell by CPE[3] (left) and immunohistochemistry (IHC, right).
(bottom) Scatter plot of immune signature score and the proportion of stromal cell calculated by 1 - CPE (left) and 1 - IHC (right).

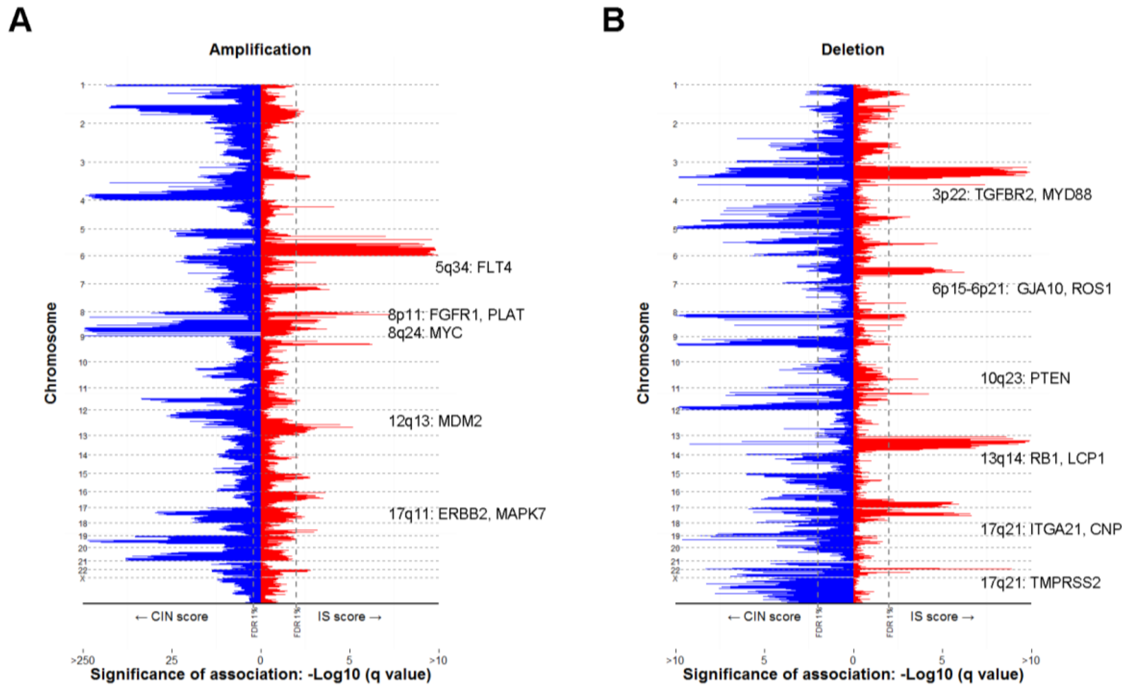

Supplementary Figure 29. Immune signature score according to chromosomal instability score. (A) The significance (FDR value) of association between amplified genes and CIN score (blue) or IS score (red). (B) The significance (FDR value) of association between deleted genes and CIN score (blue) or IS score (red). Significance is plotted according to sequence map.

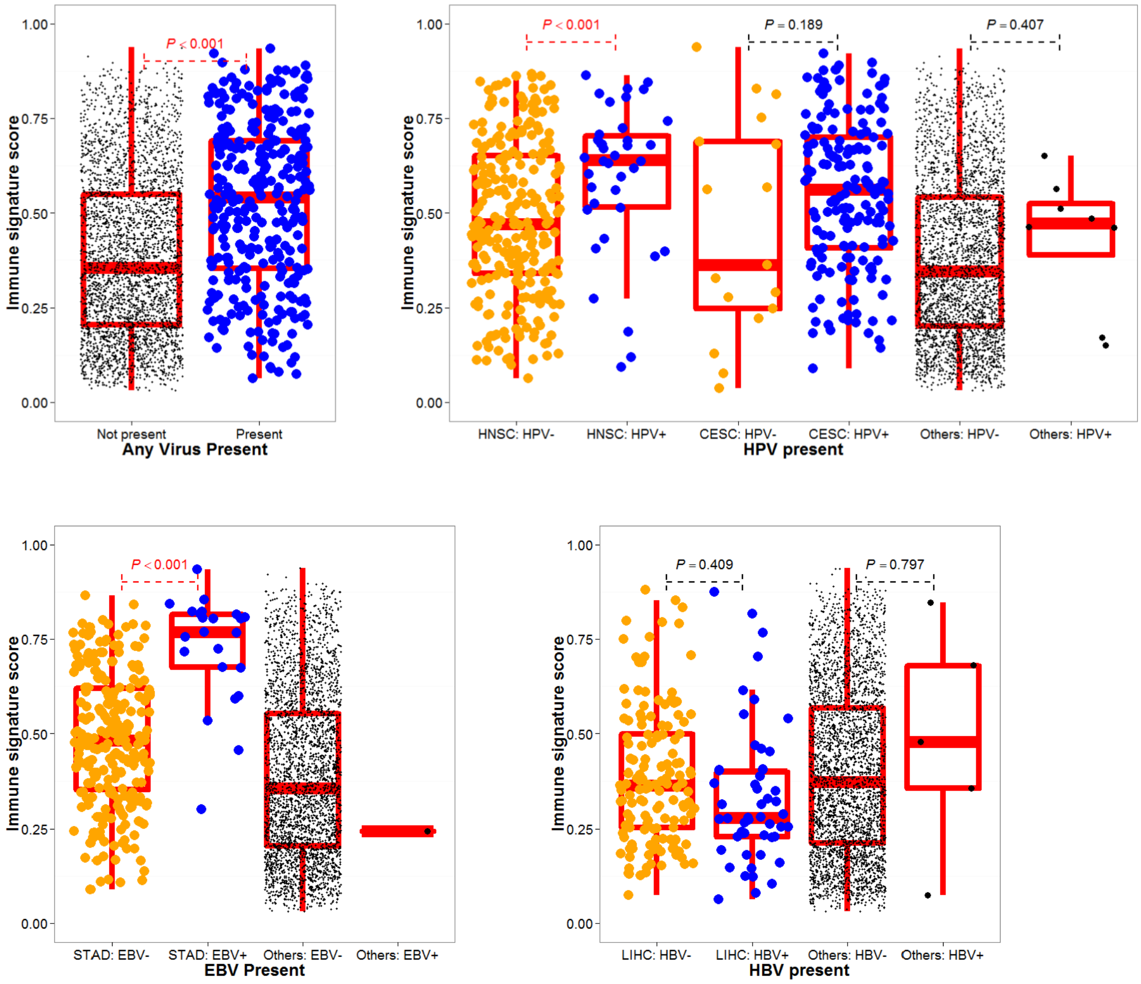

Supplementary Figure 30. Immune signature score is associated with viral presence.
Box plot of IS score according to presence of any virus (top left), Epstein-Barr virus (EBV, top right), human papillomavirus (HPV, bottom left), and hepatitis B virus (HBV, bottom right). Abbreviation: STAD, stomach adenocarcinoma; HNSC, head and neck squamous cell carcinoma; CESC, cervical & endocervical cancer; LIHC, hepatocellular carcinoma.

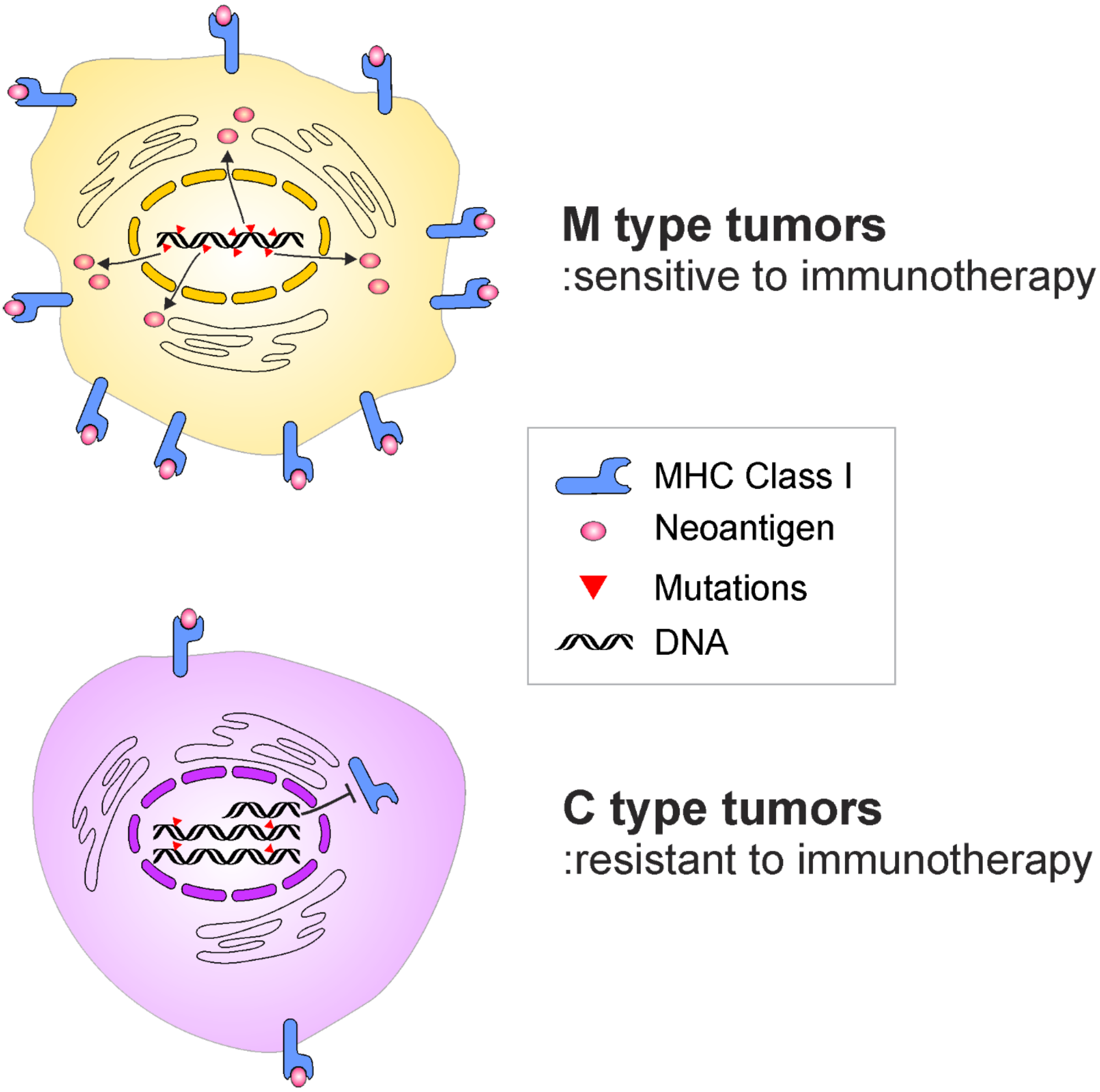

Supplementary Figure 31. Schematic diagram for M and C types of tumors.

Supplementary Reference

1. Newman AM, *et al.* Robust enumeration of cell subsets from tissue expression profiles. *Nature methods* **12**, 453-457 (2015).

2. Rooney MS, Shukla SA, Wu CJ, Getz G, Hacohen N. Molecular and genetic properties of tumors associated with local immune cytolytic activity. *Cell* **160**, 48-61 (2015).

3. Aran D, Sirota M, Butte AJ. Systematic pan-cancer analysis of tumour purity. *Nature communications* **6**, 8971 (2015).
