# Supplementary material for: Genomic landscape associated with potential response to anti-CTLA-4 treatment in cancers
Source: Nat Commun. 2017 Oct 19;8:1050. doi: 10.1038/s41467-017-01018-0 (PMC5648801; doi:10.1038/s41467-017-01018-0)
Supplement: Supplementary file 3 — Description of Additional Supplementary Files [file 41467_2017_1018_MOESM3_ESM.pdf]

Description of Additional Supplementary Files

File Name: Supplementary Data 1
Description: Genes associated with immune signature in various types of databases.

File Name: Supplementary Data 2
Description: Predicted upstream regulators of genes in immune signature score.

File Name: Supplementary Data 3
Description: Immune signature scores of TCGA tumors and data availability

File Name: Supplementary Data 4
Description: Immune signature scores and progression-free survival of immunotherapy in TCGA skin cutaneous melanoma

File Name: Supplementary Data 5
Description: Cancer genes with frequent mutation or copy number alteration

File Name: Supplementary Data 6
Description: Somatic mutations significantly associated with immune signature score

File Name: Supplementary Data 7
Description: Amplified genes significantly associated with immune signature score

File Name: Supplementary Data 8
Description: Deleted genes significantly associated with immune signature score
